# MicroRNAs as regulators of immune checkpoints in cancer immunotherapy: targeting PD-1/PD-L1 and CTLA-4 pathways

**DOI:** 10.1186/s12935-024-03293-6

**Published:** 2024-03-10

**Authors:** Arefeh Zabeti Touchaei, Sogand Vahidi

**Affiliations:** 1 Department of Chemistry, Lahijan Branch, Islamic Azad University, Lahijan, Iran; 2 Medical Biology Research Center, Kermanshah University of Medical Sciences, Kermanshah, Iran

**Keywords:** Immune checkpoint inhibitors, Immunotherapy, PD-1, PD-L1, CTLA-4, microRNA

## Abstract

Immunotherapy has revolutionized cancer treatment by harnessing the power of the immune system to eliminate tumors. Immune checkpoint inhibitors (ICIs) block negative regulatory signals that prevent T cells from attacking cancer cells. Two key ICIs target the PD-1/PD-L1 pathway, which includes programmed death-ligand 1 (PD-L1) and its receptor programmed death 1 (PD-1). Another ICI targets cytotoxic T-lymphocyte-associated protein 4 (CTLA-4). While ICIs have demonstrated remarkable efficacy in various malignancies, only a subset of patients respond favorably. MicroRNAs (miRNAs), small non-coding RNAs that regulate gene expression, play a crucial role in modulating immune checkpoints, including PD-1/PD-L1 and CTLA-4. This review summarizes the latest advancements in immunotherapy, highlighting the therapeutic potential of targeting PD-1/PD-L1 and CTLA-4 immune checkpoints and the regulatory role of miRNAs in modulating these pathways. Consequently, understanding the complex interplay between miRNAs and immune checkpoints is essential for developing more effective and personalized immunotherapy strategies for cancer treatment.

**Graphical Abstract:**

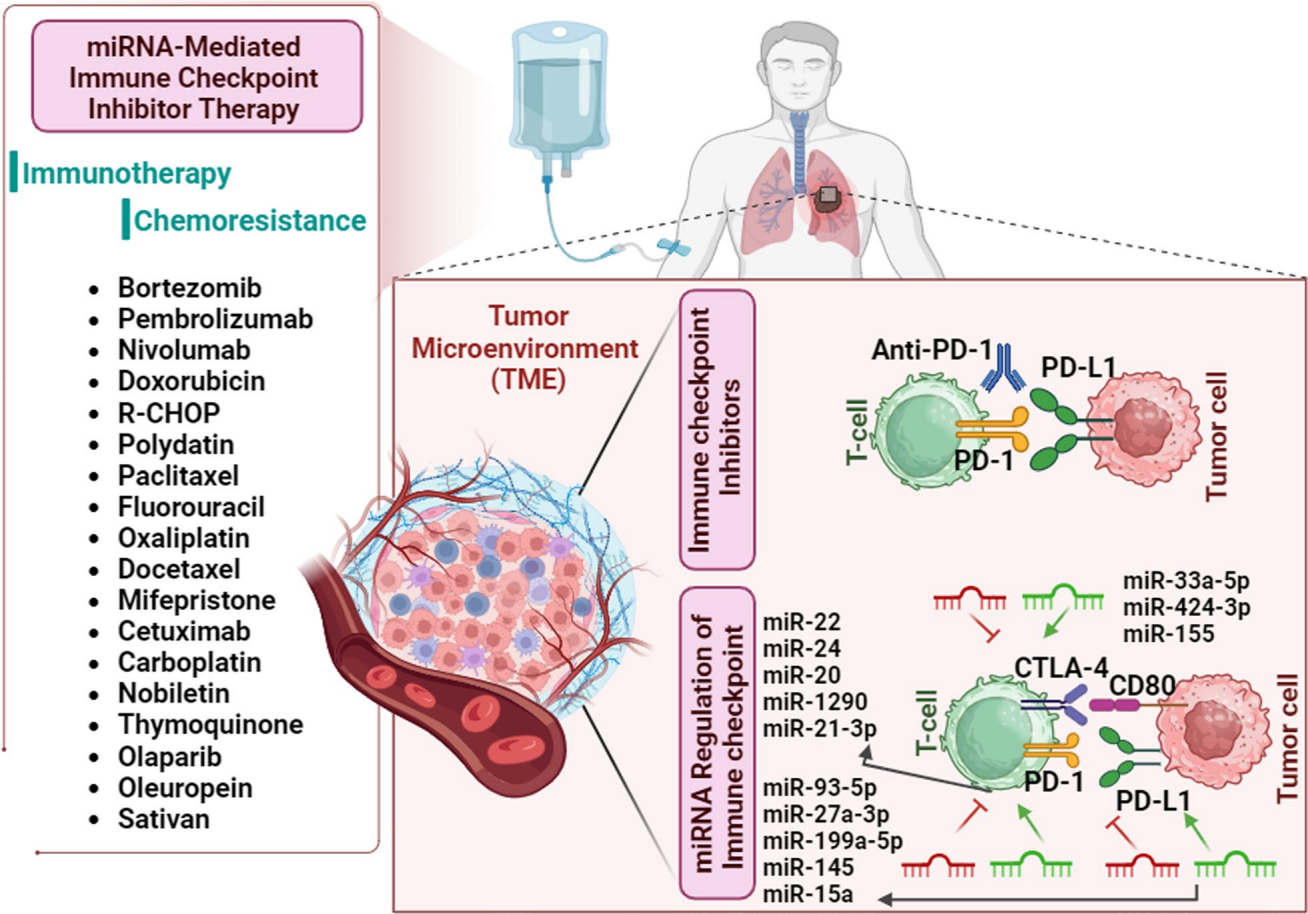

## Introduction

Cancer immunotherapies known as Immune Checkpoint Inhibitors (ICIs) focus on enhancing the body's immune response against cancer cells by targeting specific T-lymphocyte immunologic receptors. These therapies restore the balance between pro-inflammatory and anti-inflammatory signals, effectively reactivating the host immune system. Over the past decade, antibodies that target immune inhibitory receptors such as cytotoxic T lymphocyte-associated antigen 4 (CTLA-4), programmed Death 1 (PD-1), and its ligand (PD-L1) have been extensively utilized in the field of cancer treatment. Ongoing clinical development involves the investigation of various antibodies and compounds that target immune checkpoint proteins. On the other hand, the crucial role of PD-1/PD-L1 and CTLA4 as essential immune checkpoints in tumor progression is widely recognized [1–4].

### PD-1/PD-L1 inhibitors

PD-1 is a protein involved in the regulation of programmed cell death in T cells. While it is expressed on the surface of activated immune cells, it is particularly upregulated on exhausted T cells. PD-1 plays a crucial role in suppressing T-cell activity and preventing autoimmune reactions. When PD-1 interacts with its ligand, it leads to the death of cytotoxic lymphocytes in the tumor microenvironment and enhances the activity of regulatory T-lymphocytes. This activity helps the tumor evade immune control [5, 6]. Furthermore, treatments focused on inhibiting PD-1 have achieved significant success in patients diagnosed with various forms of cancer [7, 8].

PD-1 is expressed in activated T cells, B cells, and natural killer T cells. It acts as a negative regulator of T-cell activity, suppressing effector T-cell function during the effector phase. When PD-1 interacts with its ligands, PD-L1 and PD-L2, it promotes immune suppression induced by tumors [9]. On the other hand, PD-1, namely PD-L1 and PD-L2, play a role in dampening T-cell immune responses and B cell. PD-L1 is naturally expressed in myeloid cells including dendritic cells (DCs), macrophages, and myeloid-derived suppressor cells (MDSCs), at varying levels but can be induced in other cell types when exposed to pro-inflammatory stimuli. Abnormal interactions between PD-L1 and PD-1 have been linked to several diseases, such as chronic viral infections and autoimmune diseases. PD-L1 is often overexpressed in tumor cells, which can strongly inhibit anti-cancer T-cell responses in preclinical models and various types of human neoplastic diseases. Therefore, the consensus is that the overexpression of PD-L1 in tumors is often associated with disease progression and a poorer prognosis in cancer patients [10, 11]. The expression of PD-L1 in cells is controlled by the interaction of transcription factors with its promoter, which is activated by pro-inflammatory cytokines. PD-L1 is linked to an immune environment characterized by the presence of CD8 T cells, the production of Th1 cytokines and chemical factors, as well as interferos, and specific gene expression patterns [12]. One example of this process is the binding of interferon-gamma (IFN-γ) produced by T cells to the Janus kinase (JAK) signal transducer and activator of transcription (STAT) pathway. This activation leads to the transcriptional activation of interferon regulatory factor 1 (IRF1), which subsequently binds to the PD-L1 promoter and controls its expression. The tumor necrosis factor-alpha (TNFα) and IFNγ can activate the NF-κB pathway, which in turn can transcriptionally activate the expression of PD-L1. The different agents that regulate PD-L1 expression have unique functions that depend on the specific location and type of cell [13–15].

These ligands can be found in various cell types, including antigen-presenting cells (APCs) and cancer cells. When PD-1 is activated, its cytoplasmic immunoreceptors become phosphorylated, leading to the recruitment of Src homology region 2 domain-containing phosphatase-2 (SHP-2). SHP-2 then exerts inhibitory functions by suppressing intracellular molecules that participate in transmitting signals downstream of the T cell receptor (TCR) [16, 17].

PD-L1 functions as a ligand for programmed cell death, exerting inhibitory effects on tumor cells while facilitating cancer cell proliferation and migration [18]. Although some patients with elevated levels of PD-L1 expression in their tumors may experience better outcomes with immunotherapeutic drugs, it is not a reliable indicator for predicting the clinical response to treatment. Several factors contribute to this unpredictability. PD-L1 expression changes as the tumor progresses, influenced by communication between the tumor and immune system cells. This can occur due to factors such as the effects of anti-tumor therapies or the use of tyrosine kinase inhibitors that inhibit the activity of epidermal growth factor receptor (EGFR) or anaplastic lymphoma kinase (ALK). Concerning this, in immunocompetent syngeneic mouse models, the use of a tyrosine kinase inhibitor that targets EGFR, an upstream signaling molecule of PD-L1, has been shown to enhance the effectiveness of PD-1 blockade. Moreover, tyrosine kinase inhibitors, have been documented to directly or indirectly modulate the activity of glycogen synthase kinase 3 beta (GSK3β) to impact the interaction of PD-L1 [19]. It is suggested that EGFR-mutant NSCLC is highly suitable for PD-1/PD-L1 immunotherapy. Additionally, PD-L1 may serve as a promising biomarker candidate for predicting the response to EGFR-tyrosine kinase inhibitors [20]. Current research has provided evidence that EGFR-tyrosine kinase inhibitors can increase T cell infiltration in tumors, decrease the proportions of Tregs and M2-like macrophages, and improve sensitivity to α-PD-1/PD-L1 therapy in models with EGFR mutations. In summary, combining EGFR-tyrosine kinase inhibitors with α-PD-1/PD-L1 therapy has the potential to maximize the effectiveness of immunotherapy in individuals diagnosed with EGFR-mutated cancers [21].

PD-1 and its corresponding ligand, PD-L1, serve as crucial immune checkpoints that regulate immune responses. The interaction between PD-L1 expressed in cancer cells and PD-1 on immune cells promotes immune evasion by cancer cells. Consequently, inhibiting the PD-1/PD-L1 interaction is beneficial for eliminating cancer cells [22]. Blocking PD-1/PD-L1 enhances the immune response against tumors by reducing the suppressive effects of regulatory T-cells, reactivating effector T-cells, and promoting the growth of memory B-cells [23].

In early-stage tumors, the primary mechanism of antitumor effects is the inhibition of newly activated T cells from infiltrating the tumor. However, when cancer cells are introduced, apoptosis and the release of antigens occur, relying on tumor-infiltrating T cells. In patients with extensive tumor cell death or high immunogenicity, PD-1/PD-L1 blockade therapy may depend on both newly activated and reactivated T cells. Reactivating pre-existing T cells proves to be more effective in this context [24].

#### PD-1/PD-L1 interaction and immune evasion

As the PD-L1/PD-1 signaling pathway plays a significant role in tumor immune evasion, controlling the expression of PD-L1 can offer opportunities to modulate the tumor microenvironment. By inhibiting the activation of T cells, the manipulation of PD-L1 expression can disrupt the immune surveillance within the tumor microenvironment and hinder the tumor's ability to evade immune responses [25, 26]. In fact,the interaction between PD-L1 and PD-1 on immune cells triggers inhibitory responses that facilitate immune evasion and contribute to tumor progression. Excessive PD-L1 expression in numerous cancer types leads to the development of functionally exhausted and unresponsive T cells, thereby facilitating immune evasion and promoting tumor progression. Furthermore, disrupting or eliminating PD-L1 expression on tumor cells can increase the susceptibility of these cells to T cell-mediated killing [27, 28]. It has been demonstrated that treating macrophages with anti-PD-L1 antibodies results in the upregulation of mTOR pathway activity. Similarly, RNA-Seq analysis has shown the upregulation of various inflammatory pathways in macrophages following this treatment [26]. The PD-L1/PD-1 interaction can hinder T cell activation mediated by TCR (T cell receptor) by phosphorylating the intracellular tyrosine of PD-1 bound to its ligand. This phosphorylation then triggers the activation of SHP-1 and SHP-2, resulting in the dephosphorylation and deactivation of CD-3ζ and ZAP70, which are essential for TCR activation signals. Consequently, downstream signaling of the PI3K/AKT pathway is inhibited. This inhibition leads to the downregulation of Bcl-xl expression, promoting apoptosis of T lymphocytes, and suppressing the secretion of cytokines. PD-1 also activates PTEN, further inhibiting PI3K/AKT activation mediated by TCR. Additionally, it impedes the RAS-ERK1/2 signaling pathway, reducing T lymphocyte proliferation, and inhibiting IL-2 secretion by T cells [29–32].

#### PD-L1 expression modulation in radiation therapy and chemotherapy

PD-L1 expression can also be altered during radiation therapy or chemotherapy. The death of cancerous cells results in the release of a large number of antigens that are recognized by the immune system. As a result, PD-L1 expression may change in response to these treatments [23, 33, 34]. Radiation therapy affects various stages of the cancer immunity cycle, which leads to immune remodeling in the tumor microenvironment. This treatment has the potential to increase the expression of PD-L1 on cancer-associated cells as well as tumor cells. It can also raise the levels of PD-L1 in extracellular vesicles (EVs). After radiation treatment, tumor cells undergo biological processes that prompt immunomodulatory responses due to DNA damage. This damage causes dying tumor cells to release molecules known as damage-associated molecular patterns (DAMPs), and alters the immunogenicity of the irradiated tumor cells [35–37]. Radiation therapy triggers PD-L1 expression in tumor cells through four primary mechanisms, DNA damage signaling, IFN-γ signaling, cGAS-STING, and EGFR. These mechanisms are involved in the JAK-STAT pathway and are responsible for PD-L1 expression in tumor cells after radiation therapy. Radiation therapy targets solid tumors and induces DNA double-strand breaks (DSBs), the most critical type of DNA damage. PD-L1 expression is upregulated in response to DSBs in living cancer cell lines [38]. Furthermore, various proinflammatory molecules, including GM-CSF, TNF-α, LPS, I and II IFN-γ, and VEGF, stimulate PD-L1 expression. IFN-γ is a critical factor, as it stimulates downstream JAKs-STATs-IRF1 signaling, inducing PD-L1 expression. The PD-L1 promoter pathway is the IFN-γ-JAK1/JAK2-STAT1/STAT2/STAT3-IRF1 axis. Radiation therapy damages cancer cells through the generation of reactive oxygen species (ROS), which provoke inflammation responses and play a role in cell signaling [15]. In another mechanism, the cyclic GMP-AMP synthase-stimulator of interferon genes (cGAS-STING) pathway is crucial for cytosolic DNA sensing and can trigger innate immune responses [39]. As mentioned before, EGFR-TKIs can inhibit PD-L1 expression in NSCLC with mutant EGFR, and the AKT/mTOR pathway has been validated as responsible for EGFR-mediated PD-L1 expression [40].

In addition to radiation therapy, chemotherapy is a commonly used cancer treatment that can help manage the disease. However, traditional chemotherapeutic agents are usually not curative. Nonetheless, some of them have the potential to trigger immunogenic cell death and stimulate the immune system [41]. For instance, the use of Nab-paclitaxel can stimulate dying cells to release antigenic molecules, which in turn can improve the effectiveness of immunotherapy. To treat early stage of triple-negative breast cancer (TNBC), a combined treatment approach was developed that involves the administration of durvalumab, which targets PD-L1, along with nab-paclitaxel and doxorubicin/cyclophosphamide [42, 43].

Moreover, novel treatments have been developed, such as KT-1 for chemotherapy and MPPA for PD-L1 degradation immunotherapy, which demonstrate encouraging prospects for combined therapeutic strategies [44].

Scientists created a compound agent known as Gel@aPDL1 by utilizing a ROS-responsive hydrogel (PVA-SN38) and an aPD-L1 (IgG) surrogate. The hydrogel's fluorescence exhibited a slower decline compared to free IgG-Cy5.5, suggesting a sustained and controlled release of the antibody. In a mouse model with melanoma, the combined use of these two therapeutic approaches successfully suppressed tumor growth without inducing acute side effects [45]. Furthermore, a potent bifunctional antibody–drug conjugate was developed by linking MMAE to atezolizumab. This conjugate exhibited strong cytotoxicity and demonstrated a robust antitumor effect when tested in vivo [46].

### CTLA-4 inhibitor

CTLA-4, an inhibitory receptor predominantly found on T cells, undergoes upregulation following T cell activation to attenuate T cell activity. Its activation relies on stimulation through its T cell receptor (TCR) and an additional costimulatory signal, which is necessary when a naïve T cell encounters its specific antigen. Intrinsic signaling pathways lead to the recruitment of phosphatases, which inhibit transcription factors involved in T cell activity, and activate ubiquitin ligases. Additionally, extrinsically, CTLA-4 can compete with CD28 for binding to ligands, thereby reducing the overall level of costimulatory signals received by T cells. CTLA-4 is consistently present on the surface of regulatory T cells (Tregs), which have a crucial role in suppressing immune responses [47–49]. Research studies have shown that CTLA-4 regulates the function and development of Tregs. Consequently, it is presumed that both the augmentation of effector CD4 + T cell activity and the decrease in Treg cell-mediated immunosuppression are crucial components of the mechanism by which CTLA-4 blockade operates. Researchers have explored the use of CTLA-4 blockade as a potential strategy to enhance the body's immune response against tumors. CTLA-4 plays a crucial role in the regulation of activated T cells, as the absence of CTLA-4 leads to uncontrolled proliferation of T cells. In light of these newfound understandings regarding the functioning of CTLA-4, researchers sought to investigate whether blocking CTLA-4 could enhance immune responses against tumors [50–52].

It's important to note that even though Tregs and activated Tconvs (conventional T cells) both express common proteins, such as CTLA-4, there are epigenomic changes that differentiate them at the genomic level. One of these changes is Treg-specific CpG hypomethylation patterns that can be observed at the CTLA-4 locus. Various mechanisms have been proposed to explain the inhibitory effects mediated by Tregs, both in vivo and in vitro. These mechanisms include cell contact-dependent interactions and the involvement of soluble factors. Several molecules are implicated in these mechanisms, such as cell surface molecules like CTLA-4, cytokines, and various secreted or intracellular molecules. The absence or deficiency of CTLA-4 is associated with severe disease manifestations, while the presence of functional Tregs has the ability to suppress the progression of these diseases [53]. Blocking CTLA-4 in vivo using specific antibodies in mice induces autoimmune diseases, similar to the effects observed in Treg deficiency. Deletion of CTLA-4 specifically in Tregs results in systemic autoimmune and inflammatory diseases. Furthermore, CTLA-4 haploinsufficiency leads to systemic autoimmunity, likely due to compromised suppressive function of Tregs [54]. John et al. found the regulation of Tregs through B7x, a ligand from the B7 family, diminishes the effectiveness of anti-CTLA-4 treatment, which partially relies on depleting Tregs. Nevertheless, when anti-B7x and anti-CTLA-4 treatments are combined, they exhibit a synergistic therapeutic effect that overcomes the resistance caused by B7x against anti-CTLA-4 treatment [55]. In different studies, it has been observed that IL-36 promotes the expansion of Treg cells within tumor-infiltrating populations and enhances Treg proliferation. Conversely, CTLA-4 significantly enhances the antitumor effects stimulated by IL36 by depleting Tregs. The combination of CTLA-4 and IL-36 resulted in elevated proliferation and production of IFN-γ by both CD4 + and CD8 + T cells. This finding highlights a novel combination therapy that has the potential to enhance the clinical response to cancer immunotherapy [56].

The results indicate that for optimal efficacy, anti-CTLA-4 therapies necessitate both antagonism of CTLA-4 and depletion of intratumoral Tregs. However, potential future therapies that can selectively deplete Tregs in lymph nodes may exhibit efficacy even in the absence of CTLA-4 antagonism. During the clinical development of anti-CTLA-4 antibodies, immune monitoring presents both chances and challenges. CTLA-4 blockade has a significant impact on the immune response, particularly on Tregs. In initial experiments, it was demonstrated that CTLA-4 − / − T cells could be suppressed by T cells, leading to the formation of CD4 + CD25 + Tregs. A conditional knockout study in mice has shown that both conventional T cells and Tregs play a role in the development of lethal lymphoproliferative disorders. The effects of CTLA-4 blockade are mainly mediated by the effector T cell compartment, where effector T cells are critical, but Tregs are not. In order to achieve maximum anti-tumor effects, simultaneous blockade of both effector T cells and Tregs is necessary [57–59]. Enhanced specific binding of CTLA-4 to CD80 or CD86 leads to competition with CD28 for binding to important co-stimulatory molecules, inhibiting the formation of co-stimulatory signals. As a result, T cell proliferation, activation, and cell cycle progression are restrained. This leads to decreased production of cytokines and reduced expression of receptors, ultimately providing tumors with a means to evade immune surveillance [60, 61]. Research studies have demonstrated encouraging results in the use of DC-tumor fusion vaccines for various types of cancer by blocking CTLA-4. These findings suggest that targeting immune inhibitory molecules can effectively hinder T cell-mediated antitumor immune responses [62]. Notwithstanding the success of CTLA-4 monoclonal antibodies (mAb), there are several challenges associated with their use. These challenges include their large molecular weight, non-specific binding to normal tissues, heterogeneity of tumor antigens, and high production costs due to the complexity of the manufacturing process. In order to enhance the effectiveness of antitumor treatments, there is a need for new antibodies that are highly efficient and cost-effective, despite these challenges [63].

### Challenges and limitations of PD-L1 as a single biomarker for predicting ICI response

Utilizing PD-L1 expression as a biomarker for tailoring personalized immunotherapy plans and ICI treatment has gained significant importance. The primary aim of this approach is to reduce the psychological, physical, and financial burden on patients. However, relying only on PD-L1 expression as a predictive indicator has certain limitations and challenges [64, 65].

It is important to understand that PD-L1 is not a perfect biomarker. Its expression can be induced by an active immune response, so tumors with high levels of PD-L1 expression may not necessarily respond to ICIs, while tumors with low expression may still respond positively to treatment. In fact, some tumors with elevated PD-L1 expression may even demonstrate resistance to treatment. Research shows that only a minority of cases (28.9%) have a positive response to ICI treatment based on PD-L1 expression. Therefore, it is crucial to not solely rely on PD-L1 expression as a predictor of treatment response [66, 67].

Establishing standardized protocols for acquiring clinical samples, processing them, selecting antibodies, and analyzing the data is a challenging task. It is crucial to develop universally recognized procedures that can be customized for specific detection platforms, ensuring reliable and consistent results. Moreover, investigating the potential of creating more sensitive and integrated biomarkers linked with PD-L1 expression is a promising avenue, especially in various cancer types. To address these challenges, it is essential to incorporate and standardize biomarker techniques and adopt multi-biomarker strategies. These approaches can aid in selecting combination ICI therapies, thus expanding the clinical advantages of immunotherapy to suitable subgroups of patients [68, 69].

Researchers are currently working on discovering new markers or combinations of markers that can provide more accurate information to predict the effectiveness of anti-PD-L1 immunotherapy. They are also exploring real-time tracking of immune cells and non-invasive methods for evaluating the response of tumors to immunotherapy. Furthermore, blood-based tests are being investigated to identify relevant factors that can determine the clinical efficacy of immunotherapy. Additionally, researchers are developing in vitro research models, such as organoids, which include the immune microenvironment, to provide a more precise representation of tumor progression and drug resistance, and assist in the screening of anticancer drugs [70, 71].

It is possible to improve the effectiveness of PD-1 blockade therapy through the development of combination treatments with other checkpoints such as CTLA-4 and NKG2D. Additionally, exploring molecular markers like PD-L1, TMB, MSI, and TIL shows promise in increasing therapy efficacy. The use of single-cell sequencing technology is proving to be valuable in understanding the heterogeneity of the tumor microenvironment, offering crucial insights into tumor development and the mechanisms of immune evasion [72, 73].

In summary, PD-L1 expression has been widely used as a biomarker to predict clinical responses to immunotherapy. However, it is important to recognize its limitations and tackle the challenges associated with it. Improving standardization, exploring alternative biomarkers, and advancing diagnostic techniques are crucial for enhancing the effectiveness and applicability of immunotherapy in the treatment of cancerous tumors [66].

## MicroRNAs: bridging the gap between gene regulation, immune checkpoints, and cancer management

MicroRNAs (miRNAs) are molecules involved in post-transcriptional gene regulation, exerting their function by directly binding to and inhibiting the translation of human mRNA. The regulatory role of microRNAs in gene expression has been well-documented and plays a significant role in various aspects of cancer biology [74]. MiRNAs are encoded in various parts of the genome, such as intergenic regions or within the introns or exons of protein-coding genes. Following transcription, cleavage, and processing, mature miRNAs are typically transported from the nucleus to the cytoplasm, where they are loaded into the RNA-induced silencing complex (RISC) [75]. Due to the undeniable association between abnormal gene expressions and the initiation and progression of cancer, miRNA-based therapy has emerged as a promising option for the treatment of this disease [76]. miRNAs have also been implicated in the regulation of immune checkpoints, specifically in the case of PD-1 and PD-L1. In addition, Gene expression, particularly the role of long non-coding RNAs (lncRNAs), plays a significant role in the therapy of cancer [77–79]. Hence, miRNAs show significant potential as diagnostic biomarkers and therapeutic targets for managing cancer complications associated with ICIs.

Tumor-suppressor miRNAs play a role in controlling the antitumor immune response by regulating immune checkpoints such as PD-1, PD-L1, and CTLA-4. A set of miRNAs functions to shield cancer cells from immune clearance by reducing the immunogenicity of cancer cells and suppressing the magnitude of the anti-cancer immune response. In contrast, another group of miRNAs enhances the immune clearance of cancer cells. These miRNAs, which modulate the immune response, are referred to as im-miRNAs [80]. Certain miRNAs target either PD-1 or PD-L1 checkpoint proteins, while others simultaneously target both PD-1 and PD-L1. miRNAs also play a role in regulating crucial immune cells present in the tumor microenvironment, including macrophages, MDSCs, and natural killer (NK) cells. Additionally, miRNAs are involved in regulating tumor antigen processing for the presentation on the major histocompatibility complex (MHC). The abnormal expression of miRNAs in tumors can influence various pathways related to cancer progression and metastasis. Depending on the target gene, a miRNA can function as an onco-miR, promoting tumor growth, or as a tumor-suppressor miRNA, inhibiting cancer development. While the expression of PD-1/PD-L1 is commonly associated with immune evasion, intriguingly, several cancer studies have reported an emerging role of PD-1/PD-L1 in cancer development and progression [81].

Overall, miRNAs have the potential to serve as a crucial element in the series of biomarkers that can predict the efficacy of immune checkpoint inhibitors in tumor response. The assessment of miRNAs in plasma samples before and after treatment offers a convenient means of evaluating their potential as noninvasive biomarkers for monitoring the response to immune checkpoint inhibitor therapy [82]. As mentioned in the following review, it has been demonstrated that miRNAs can interact with the ICs gene in specific types of cancer but not in others. Conversely, there are instances where a particular miRNA can interact with the ICs in multiple types of cancer [83, 84]. The findings from both in vitro and in vivo experiments investigating the potential of miRNAs as immunotherapeutic agents are promising. However, in order to utilize miRNA-based drugs in clinical settings, they must meet efficacy and safety standards. Employing techniques that enhance the specificity of miRNA binding to their targets can improve the effectiveness of miRNA-based therapy and potentially reduce the required drug dosage, thereby minimizing associated side effects [85–87].

### microRNA regulation of PD-1 in cancer

miRNAs have emerged as important regulators of immune checkpoint pathways, including PD-1, in various types of cancer. The Table 1 provides a summary of the microRNA regulation of PD-1 in various cancer types, including melanoma, NSCLC, HCC, GC, and triple-TNBC. It highlights the specific microRNAs involved, their targets, the underlying mechanisms, and their effects on PD-1 expression and response to anti-PD-1 immunotherapy.

**Table 1:** microRNA regulation of PD-1 in different cancer types

Cancer type	MicroRNA	Target	Mechanism
Melanoma	miR-22, miR-24	PD-1	Negative correlation with plasma PD-1 levels in responders
	miR-1290		Inverse correlation with CD3-negative, CD8-negative, and PD-1-positive cells
	miR-100-5p, miR-125b-5p		Upregulation linked to better results with anti-PD-1 therapy
	miR-21-3p		Over expressed by ATF3, increases anti-PD-1 immunotherapy effectiveness
NSCLC	hsa-miR-320	PD-1	Upregulated associated with negative response to PD-1 inhibitors
	miR-125b-5p		Downregulated in positive responders to anti-PD-1 therapy
	miR-30a-5p		hsa_circ_0020714 influences the miR-30a-5p/SOX4 axis, leading to the promotion of immune evasion and resistance against anti-PD-1 therapy
HCC	miR-497-5p	PD-1	CircSOD2 functions as a sponge for miR-497-5p, thereby promoting immune evasion and resistance to anti-PD-1 therapy
	miR-15a-5p		Suppresses HCC proliferation, migration, and aggression, inhibits PD-1 expression
GC	miR-940	PD-1	Regulates PD-1 checkpoint pathway, promotes cell migration
TNBC	miR-185-5p	PD-1	CircFGFR4 influences the miR-185-5p/CXCR4 axis, promotes immune evasion and resistance to anti-PD-1 therapy
	miR-149-3p		Downregulates T-cell inhibitor receptors (including PD-1) and T-cell exhaustion, enhances T-cell activation

#### MicroRNA regulation of PD-1 in melanoma

The presence of a negative relationship between the levels of *miR-22* and *miR-24* and plasma PD-1 levels in patients who responded well to treatment for an extended period suggests that a network of miRNAs may play a role in suppressing the expression of immune checkpoints, primarily through the involvement of the *miR-20* family (p < 0.05) [88]. The levels of *miR-1290* in circulating plasma exhibited an inverse correlation with the presence of CD3-negative, CD8-negative, and PD-1-positive cells [89, 90]. In a group of melanoma biopsies that had not been treated with PD-1 inhibitors, it was observed that higher levels of *miR-100-5p* (p-value = 0.036) and *miR-125b-5p* (p-value = 0.025) expression were linked to better results when treated with anti-PD-1 immunotherapy [91]. The upregulation of *miR-21-3p* induced by *ATF3* played a role in enhancing the effectiveness of anti-PD-1 immunotherapy in melanoma. This upregulation facilitated tumor cell ferroptosis by suppressing the novel target *TXNRD1* and promoting lipid peroxidation within the tumor (p < 0.05) [92].

#### miRNA regulation of PD-1 in non-small cell lung cancer (NSCLC)

The expression of *hsa-miR-320* family was increased and linked to a negative response to PD-1 inhibitors in non-small cell lung cancer (NSCLC). Conversely, *miR-125b-5p* expression decreased in patients who responded positively to anti-PD-1 therapy (p < 0.05) [93]. *CircFGFR1*, a significant gene in NSCLC tissues, plays a crucial role in cell proliferation, migration, invasion, and immune evasion. Interestingly, the knockout of CXCR4 resulted in enhanced sensitivity of NSCLC cells to anti-PD-1 immunotherapy. *CircFGFR1* functions as a miRNA sponge for *miR-381-3p*, leading to the upregulation of *CXCR4* expression. This upregulation promotes the progression of NSCLC and contributes to resistance against PD-1-based therapy [94].

*hsa_circ_0020714* promotes immune evasion and resistance to anti-PD-1 immunotherapy in NSCLC by modulating the *miR-30a-5p/SOX4* axis. *hsa_circ_0020714* acts as a natural sponge for *miR-30a-5p*, leading to increased expression of *SOX4*. This mechanism ultimately facilitates immune evasion and resistance to anti-PD-1 therapy in patients with NSCLC [95].

#### miRNA regulation of PD-1 in hepatocellular carcinoma (HCC)

*CircSOD2* is upregulated in Hepatocellular carcinoma (HCC), and its knockdown leads to cell growth inhibition, apoptosis promotion, cell cycle arrest, and metastasis suppression. *CircSOD2* promotes HCC development by acting as a miR-497-5p sponge, targeting *ANXA11* and promoting immune evasion and anti-PD-1 resistance [96]. In a particular study, the overexpression of *miR-15a-5p* was found to suppress the proliferation, migration, and aggression of HCC. Moreover, *exo-miR-15a-5p* released by CD8 + T cells directly targeted and inhibited PD-1 expression. Subsequently, when PD1-transfected CD8 + T cells were co-cultured with *miR-15a-5p*-transfected HCC cells, it was observed that PD-1 diminished the inhibitory effects of *miR-15a-5p* on HCC progression (p < 0.05) [97].

#### miRNA regulation of PD-1 in gastric cancer (GC)

In gastric cancer (GC), *miR-940* is involved in the regulation of the PD-1 checkpoint pathway via the *CBL-b/Stat5a* axis, which ultimately promotes cell migration [98].

#### miRNA regulation of PD-1 in triple-negative breast cancer (TNBC)

The circular RNA *circFGFR4* plays a crucial role in immune evasion and resistance to anti-PD-1 immunotherapy in TNBC by modulating the *miR-185-5p/CXCR4* axis. Elevated levels of *circFGFR4* were associated with reduced infiltration of CD8 + T cells in tumor tissues and resistance to anti-PD-1 immunotherapy in both TNBC patients and mice with TNBC tumors [99]. As well as, the binding of *miR-149-3p* to the 3'UTRs of mRNAs that encode T-cell inhibitor receptors such as PD-1, TIM-3, BTLA, and Foxp1 was predicted in breast cancer. Several effects were observed when CD8 + T cells were treated with a mimic of *miR-149-3p*. Apoptosis was reduced, and changes in mRNA markers indicated decreased T-cell exhaustion. The mRNAs encoding PD-1, TIM-3, BTLA, and Foxp1 were downregulated. On the other hand, T-cell activation was increased, as evidenced by enhanced proliferation and secretion of effector cytokines such as IL-2, TNF-α, and IFN-γ, after treatment with the *miR-149-3p* mimic [100] (p < 0.05).

**Fig. 1:**
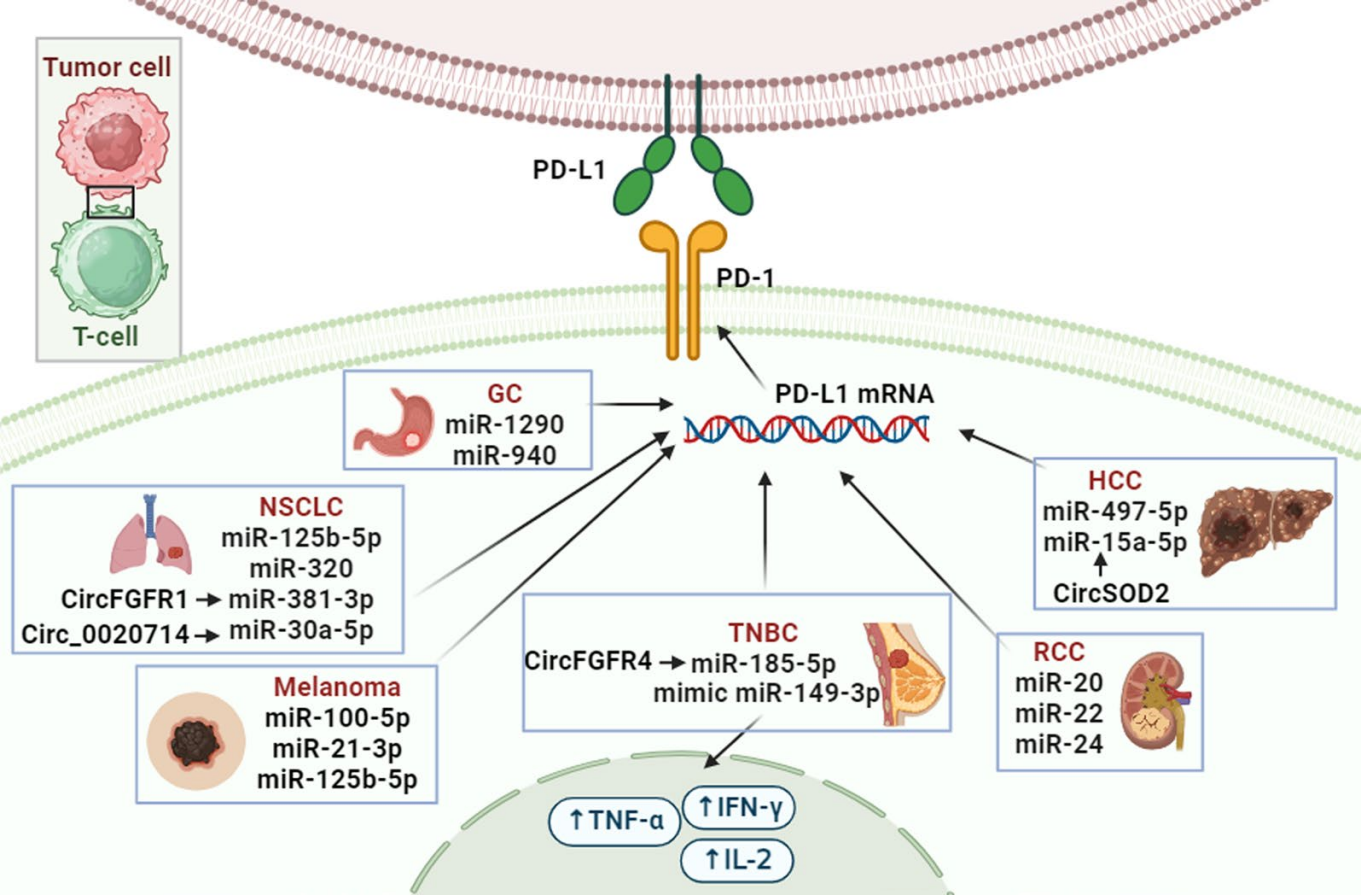
The regulatory relationships between different miRNAs and PD-1 expression in various cancer types. Figure provides an overview of the microRNA regulation of PD-1 in melanoma, NSCLC, HCC, GC, and TNBC. It highlights the specific microRNAs involved, their targets, underlying mechanisms, and their effects on PD-1 expression. *NSCLC* non-small cell lung cancer, *GC* gastric cancer, *TNBC* triple-negative breast cancer, *HCC* hepatocellular carcinoma, *RCC* renal cell carcinoma

Figure 1 depicts the regulatory relationships between specific miRNAs and the expression of PD-1, an immune checkpoint protein, in various cancer types including gastric cancer (GC), hepatocellular carcinoma (HCC), non-small cell lung cancer (NSCLC), melanoma, renal cell carcinoma (RCC) and triple-negative breast cancer (TNBC). The arrows indicate the regulatory interactions, with each arrow representing the downregulation or upregulation of PD-1 expression by the corresponding miRNA. In summary, Fig. 1 highlights the diverse roles of miRNAs in modulating PD-1 expression, potentially influencing immune evasion and response to anti-PD-1 immunotherapy in different cancer types.

### microRNA regulation of PD-L1 in cancer

Certain miRNAs control the expression of PD-L1 and have promising applications in therapy. There was an inverse correlation observed between *miR-200c-3p* and the expression of immune cell markers, including CD3, CD4, and CD8. Additionally, *miR-200c-3p* displayed a negative correlation with the presence of PD-L1, an immune marker [89, 101]. In the initial stage of clinical trials, the utilization of *MRX34*, a newly developed synthetic version of *miR-34a-5p*, has demonstrated a notable reduction in the expression of PD-L1 in tumors. Furthermore, when *MRX34* was combined with radiotherapy, it increased the number of CD8 + cells and decreased the infiltration of tumors by macrophages and Treg cells (p < 0.05) [102].

#### miRNA-mediated regulation of PD-L1 in lung cancers

*miR-200/ZEB* pathway is closely associated with increased PD-L1 expression, reduced infiltration of CD8 + cells, and higher scores for epithelial-mesenchymal transition (EMT). Additionally, researchers have observed significantly higher levels of PD-L1 expression in mesenchymal lung cancer cells compared to lung carcinoma cells [103]. In a mouse model, *miR-200* inhibited the EMT process by directly targeting PD-L1, which resulted in a delay in the progression of cancer. Additionally, there was an inverse relationship observed between the expression of *miR-200* and PD-L1, indicating the possibility of using miRNA expression as a predictive biomarker for assessing the response to immunotherapy [103].

The expression of PD-L1 is inversely related to *miR-197-5p* through the CDC28 protein kinase regulatory subunit 1B (CKS1B)/STAT3 pathway. Additionally, *miR-197-5p* is linked to a shorter survival rate in patients with NSCLC (p < 0.05) [104].

*miR-138* functions as a tumor suppressor by inhibiting the proliferation and migration of tumor cells, and it also plays a role in regulating immune response. Nevertheless, treatment with *miR-138-5p* resulted in reduced growth of tumor cells and an increased presence of dendritic cells (DCs) within the tumor. In addition, *miR-138-5p* was discovered to specifically target PD-L1, leading to the downregulation of PD-L1 expression on tumor cells. This downregulation resulted in a decrease in the expression of Ki67 and minichromosome maintenance (MCM) in tumor cells, indicating reduced proliferation. Furthermore, the decreased expression of PD-L1 also reduced the tolerance effect on DCs. *miR-138-5p* also directly lowers the expression of PD-L1 on DCs and PD-1 on T cells. Similar findings were observed in both isolated NSCLC cells and DCs. As a result, *miR-138-5p* not only inhibits tumor growth but also activates the immune system by downregulating PD-1/PD-L1. These findings highlight the potential of *miR-138-5p* as a promising therapeutic target for treating NSCLC (p < 0.05) [83]. Moreover, In NSCLC, *miR-940* upregulates the expression of PD-L1 by inhibiting c-Cbl, thereby promoting the activation of the STAT3/AKT/ERK signaling pathway. PD-L1 expression is elevated in association with EGFR mutations, ALK rearrangements, and KRAS mutations. In other words, the upregulation of PD-L1 is connected to the activation of STAT3, AKT, and ERK. Furthermore, *miR-940* could potentially regulate Cbl-b and c-Cbl in NSCLC cells. Cbl-b and c-Cbl suppress the expression of PD-L1 by reducing the activity of the p-STAT3, p-AKT, and p-ERK pathways. NSCLC samples show a negative association between Cbl-b/c-Cbl and PD-L1; these proteins are linked to overall survival. During T-cell activation, the expression of Cbl-b increases due to PD-1/PD-L1 signaling. On the other hand, in tumor cells, both Cbl-b and c-Cbl inhibit PD-L1, which helps to alleviate immune suppression. These discoveries underscore the significance of Cbl proteins in regulating immune responses [105–107].

The confirmation of the immune evasion mechanism involving *SOX2-OT* and the stimulating impact of the *SOX2-OT/miR-30d-5p/PDK1* axis on PD-L1, operating via the mTOR signaling pathway, has been demonstrated in NSCLC [108]. In addition, *PSMA3-AS1* plays a role in enhancing the growth, movement, and invasion of NSCLC cells by controlling the activity of *miR-17-5p* and PD-L1. *PSMA3-AS1* acts by competitively attaching to *miR-17-5p*, which would otherwise regulate PD-L1. In lung cancer cells, the level of *miR-17-5p* is typically low, while the expression of PD-L1 is high [109]. *Circ_0000284*, a circular RNA, was identified as a target of *miR-377-3p*, as well as PD-L1. Furthermore, it was determined that *Circ_0000284* acts as a cancer-promoting molecule in NSCLC by modulating the *miR-377-3p*/PD-L1 pathway. By functioning as a competing endogenous RNA (ceRNA) for miR-377, *circ_0000284* up-regulates the expression of PD-L1, thereby promoting the progression of NSCLC (p < 0.05) [110].

In NSCLC, both *CircFOXK2* and PD-L1 levels were increased. *CircFOXK2* acted as a regulator of NSCLC tumorigenesis and the cytotoxicity of CD8 + T cells by targeting *miR-485-5p*, which in turn could bind to PD-L1. The lack of *circFOXK2* had inhibitory effects on NSCLC tumorigenesis and CD8 + T cell cytotoxicity, which were compromised by *miR-485-5p* inhibition or PD-L1 overexpression [111]. Furthermore, *CircCHST15* facilitated the immune escape of lung cancer cells by acting as a sponge for miR-155-5p and miR-194-5p, consequently promoting the expression of PD-L1. *CircCHST15* specifically targeted *miR-155-5p* and *miR-194-5p*, with the latter further regulating PD-L1 expression, exhibiting a positive correlation. Notably, *CircCHST15* contributed to tumor growth, resulting in the downregulation of *miR-155-5p* and *miR-194-5p* and the upregulation of PD-L1 expression [112].

The antitumor immune response of *miR-4458* in NSCLCs revealed a decrease in *miR-4458* expression and an increase in *STAT3* levels in NSCLCs. Through in vitro experiments, it was observed that *miR-4458* inhibits cell proliferation and reduces the expression of PD-L1. Furthermore, it was confirmed that *STAT3* is a target gene of *miR-4458*, and increasing *STAT3* levels enhances its ability to suppress cell proliferation and PD-L1 expression. In animal studies, it has been demonstrated that the overexpression of *miR-4458* impedes tumor growth, reduces the presence of PD-1 + T cells, and increases the levels of IFN-γ and IL-2 [113].

Besides, the targeting of PD-L1 by *miR-200a-3p* has been verified, and its regulation is indirectly influenced by *MALAT1*. In summary, the *MALAT1* promotes the progression of NSCLC by modulating the *miR-200a-3p*/PD-L1 axis [114].

**Fig. 2:**
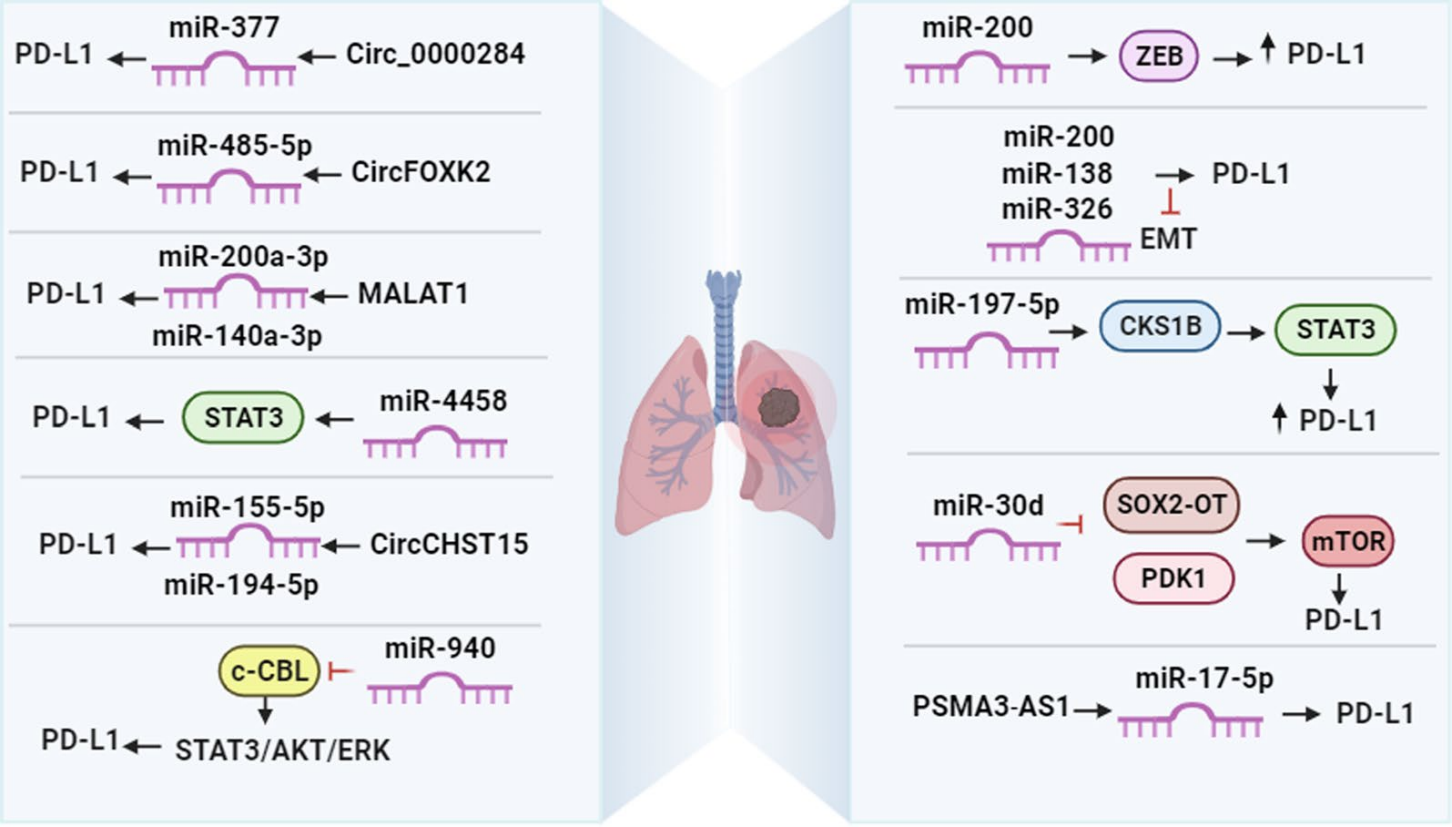
The regulatory roles of different miRNAs in the expression of PD-L1 in lung cancers

In lung adenocarcinoma (LUAD), *miR-326* has been identified as a suppressor of immune checkpoint molecule PD-L1 expression. Consequently, this leads to alterations in the cytokine profiles of CD8 + T cells and a reduction in tumor cell migration. By downregulating PD-L1 expression and enhancing T cell cytotoxic function, *miR-326* acts as a restraining factor in tumor progression for LUAD. Conversely, the decreased expression of *miR-326* promotes the migration of tumor cells. These findings indicate a potential novel approach for utilizing *miR-326* in tumor immunotherapy [115]. *MALAT1* has the potential to act as a molecular sponge for *miR-140a-3p*, leading to an increase in PD-L1 expression and a reduction in the sensitivity of LUAD cells to radiation. Additionally, when *MALAT1* was suppressed, it resulted in the inhibition of PD-L1 mRNA and protein expressions in LUAD cells by upregulating *miR-140* (p < 0.05) [116] **(**Fig. 2**)**.

#### miRNA-mediated regulation of PD-L1 in gastrointestinal cancers

##### Colorectal cancer

PD-L1 is known to be an important target for *miR-214* in colorectal cancer. The interaction between axis circEIF3K/miR-214/PD-L1 plays a role in facilitating the advancement of colorectal cancer under hypoxic conditions through the involvement of cancer-associated fibroblasts (CAFs). *circEIF3K* exosome is secreted under hypoxia. exosome from *circEIF3K* knockdown CAF significantly attenuated proliferation, invasion and tube formation of colorectal cancer cells, which could be reverted by *miR-214* under hypoxia treatment [117]. Correspondingly, *miR-140-3p* functions as a cancer-inhibiting factor in CRC through its direct targeting of PD-L1 and the subsequent inactivation of the PI3K/AKT pathway. In CRC tissues, there is an elevated expression of PD-L1, which is negatively associated with the expression of *miR-140-3p*. The inhibition of PD-L1 results in comparable biological patterns in CRC cells, and when *miR-140-3p* is partially restored, it partially alleviates its inhibitory effects [118]. The *lncRNA KCNQ1OT1* found in exosomes released by tumor cells has the ability to control the ubiquitination of PD-L1 through the miR-30a-5p/USP22 pathway, thereby facilitating immune evasion in CRC [119].

*LINC01088*, another long non-coding RNA, was found to be significantly upregulated in CRC. It primarily localized in the cytoplasm and was observed to directly target *miR-548b-5p* and *miR-548c-5p*. This interaction resulted in the upregulation of Ras GTPase-activating protein-binding proteins 1 (G3BP1) and PD-L1 expression, leading to alterations in CRC cell phenotypes. Besides, the knockdown of *LINC01088* suppressed CRC tumor growth and metastasis. The inhibitory effects on tumor growth due to *LINC01088* knockdown were reversed by overexpression of G3BP1 [120].

A strong connection was discovered between PD-L1 and IFNγ, as well as elevated levels of both PD-L1 and IFNγ, along with *miR-93-5p*, in samples taken from both the tumor and margin areas of colorectal cancer. Since colorectal cancer is a significant contributor to the expression of PD-L1, IFNγ, and *miR-93-5p*, by comprehending the mechanisms that drive PD-L1 expression within the tumor, new prospects may emerge for focused immunotherapy in the treatment of CRC (p values < 0.05) [121]. miR-148a-3p has been identified as a potential suppressor of PD-L1 expression in CRC. It directly interacts with the 3'-UTR of the PD-L1 gene, resulting in decreased levels of PD-L1 in both the whole cell and cell surface. Additionally, the overexpression of *miR-148a-3p* reduces the expression of PD-L1 on tumor cells induced by IFNγ and reduces T-cell apoptosis [122].

A study conducted by Tian et al. found that CRC shows abnormal expression of *SETDB1*, which is positively correlated with the expression of PD-L1. The study discovered that *SETDB1* negatively regulates the expression of *miR-22* by downregulating *FOSB*. Moreover, *SETDB1* was found to downregulate PD-L1 expression by targeting *BATF3*. When *SETDB1* was silenced, T cell-mediated cytotoxicity against tumor cells increased, leading to hindered tumor growth and reduced infiltration of immune cells (p values < 0.05) [123].

In CRC, the levels of *hsa_circ_0136666* and PD-L1 are increased, while the level of *miR-497* is decreased. *Hsa_circ_0136666* directly interacts with *miR-497* and regulates PD-L1 expression by binding to the 3' UTR of PD-L1. Through this mechanism, *hsa_circ_0136666* controls cell proliferation and apoptosis by targeting *miR-497* and modulating PD-L1 expression. Moreover, *hsa_circ_0136666* stimulates the activation of Treg cells via the *miR-497*/PD-L1 axis and its downstream signaling pathway. Overall, *hsa_circ_0136666* promotes the expression of PD-L1 by suppressing *miR-497* levels in CRC, leading to the activation of Treg cells and immune evasion [124].

*miR-124* is significantly downregulated in CRC tissues and shows a negative correlation with PD-L1 expression. When CRC cells were transfected with *miR-124* mimics, there was a decrease in PD-L1 mRNA, protein, and cell surface expression. This leads to inhibition of Tregs and reduced cell proliferation. In addition, *miR-124* overexpression leads to decreased colony and spheroid formation ability, decreased matrix metalloproteinase 9 (MMP-9) expression, and suppression of cell migration and invasion [125].

In CRC, tumor-associated macrophages (TAMs) in the tumor microenvironment are abundant and express high levels of PD-L1. The CRC cells release small extracellular vesicles (sEVs), which promote M2-like polarization of TAMs and enhance PD-L1 expression. As a result, there is an increase in macrophage abundance and a decrease in T cell activity. These effects are mediated by CRC-derived *miR-21-5p* and *miR-200a*, which lead to reduced CD8 + T cell activity and enhanced tumor growth. Targeting the inhibition of sEV-miRNA secretion and PD-L1 in TAMs could offer a novel treatment strategy and sensitize CRC to anti-PD-L1 therapy [126].

Liu et al. observed a correlation between PD-L1 mRNA levels and the infiltration of CD8 + T cells, as well as improved prognosis. They discovered that *miR-15b-5p* can downregulate the expression of PD-L1, leading to the inhibition of tumor formation and increased sensitivity to anti-PD-1 treatment in models of colitis-associated cancer (CAC) and CRC. Additionally, the researchers found that IL-17A can induce high levels of PD-L1 expression in CRC cells by regulating the *P65/NRF1/miR-15b-5p* pathway. The combination of IL-17A and PD-1 blockade exhibited efficacy in CRC tumors, resulting in increased presence of cytotoxic T lymphocytes and decreased presence of myeloid-derived suppressor cells within the tumors [127].

Furthermore, several microRNAs, including *miR-191-5p*, *miR-382-3p*, *miR-200a-3p*, *miR-200c-3p*, and *miR-138-5p* have demonstrated the ability to suppress PD-L1 expression in CRC cells. These microRNAs exhibit various beneficial effects, such as reducing tumor migration, promoting anti-tumoral immune responses, inhibiting tumor growth, decreasing tumor cell viability, and enhancing sensitivity to chemotherapy (p values < 0.05) [128].

##### Gastric cancer

In gastric cancer, a specific cis-acting region consisting of just 100 nucleotides was identified as crucial for the post-transcriptional repression of PD-L1 expression. Dysregulation of *miR-105-5p*, which is commonly observed in various cancer types, was predicted to bind to this region and exhibit a negative correlation with PD-L1 expression. When *miR-105-5p* was overexpressed in gastric cancer cells, it resulted in reduced PD-L1 expression and triggered the activation of CD8 + T cells [129]. Besides, In GC, *miR-940* has the ability to target CBL-b, resulting in the promotion of *STAT5A* ubiquitination and the inhibition of PD-L1 expression [98]. *miR-429* directly targeted PD-L1, and its overexpression inhibited the development of GC by suppressing PD-L1. Furthermore, the long non-coding RNA hypoxia-inducible factor-1 alpha antisense RNA-2 (*HIF1A-AS2*) indirectly regulated PD-L1 expression by acting as a sponge for *miR-429* [130].

*MiR-502-5p* targeting PD-L1 has emerged as a promising therapeutic target for GC treatment, as it inhibits tumor growth and metastasis. Additionally, *miR-502-5p* hinders the aggressive characteristics of GC by downregulating PD-L1 expression both at the transcriptional and post-transcriptional levels [131].

Surgical removal of gastric cancer has an impact on the equilibrium between Th17 and Treg cells, leading to an increase in PD-1 and PD-L1 expression. In laboratory tests conducted in vitro, the introduction of Ad-sh-PD1 through transfection helped mitigate the imbalance between Th17 and Treg cells to some extent, achieved in part by upregulating the expression of *miR-21*. Consequently, the upregulation of PD-1/PD-L1 expression has been demonstrated to lead to a reduction in Th17 cell population and an augmentation in Treg cell population. This alteration is accompanied by a decrease in the expression of RORγt and IL-17, while there is an increase in the expression of *Foxp3* and TGF-β1 [132].

GC is associated with a high expression of *hsa_circ_0136666*. This particular circular RNA has been found to promote tumor proliferation and the formation of a tumor microenvironment, which ultimately leads to immune evasion. Importantly, CD8 + T cells are involved in this process. *Hsa_circ_0136666* upregulates the expression of PRKDC by sponging *miR-375-3p* in a competitive manner, which in turn regulates immune checkpoint proteins and phosphorylates PD-L1. This process causes the aggregation of PD-L1 and suppresses immune function, leading to impaired cancer immune responses [133]. In both in vitro and in vivo experiments, contrasting effects were observed when miR-375 was knocked down. According to luciferase reporter assays, *miR-375* can bind to the 3'-UTR regions of JAK2. Also, a negative correlation was found between the expression of *miR-375* and JAK2/STAT3/PD-L1 in GC cell lines [134].

According to a study, exosomes obtained from M1 macrophages carrying *miR-16-5p* exhibited the ability to induce a T cell immune response and impede tumor formation in both laboratory experiments and animal models. This was accomplished by reducing the expression of PD-L1 [133].

The *circSCUBE3/miR-744-5p*/SLC7A5 axis has been found to play a role in immune evasion and resistance to anti-PD-1 treatment in GC. In GC, *hsa_circ_0076092* is upregulated and associated with a poor prognosis, which can suppress immune escape by regulating PD-L1. More specifically, *circSCUBE3* binds to *miR-744-5p*, which targets SLC7A5 [135].

PD-L1 was targeted by *miR-5193*, *miR-4443*, *miR-520 h*, and *miR-496* in GC. As well, Overexpression of *miR-5193* in GC cells leads to reduced expression of PD-L1, which enhances the anti-tumor activity of T cells. This indicates that targeting low levels of *miR-5193* in the bloodstream through nucleic acid immunotherapy could be a potential treatment for GC [136].

##### Pancreatic cancer

The *PSMB8-AS1/miR-382-3p/STAT1*/PD-L1 axis shows potential as a viable therapeutic target in pancreatic cancer. *PSMB8-AS1* enhances the proliferation and metastasis of pancreatic cancer cells by acting as a sponge for *miR-382-3p*, leading to an upregulation of *STAT1* expression. Ultimately, *STAT1* transcriptionally activates PD-L1 and contributes to its stability [137].

Similarly, a high level of *miR-194-5p* expression in individuals with pancreatic cancer is associated with improved survival rates. Conversely, an increased expression of PD-L1 is linked to poorer survival rates. There is a negative correlation between PD-L1 expression and *miR-194-5p* expression, and *miR-194-5p* specifically targets PD-L1. When *miR-194-5p* is overexpressed, it hinders the migration, invasion, and proliferation of pancreatic cancer cells. In a mouse model, *miR-194-5p* inhibits the progression of pancreatic cancer, facilitates the infiltration of CD8 + T cells, and enhances the production of IFN-γ. Therefore, targeting *miR-194-5p* could be a promising approach for immunotherapy in pancreatic ductal adenocarcinoma by suppressing the expression of PD-L1 [138]. According to the research, *miR-142-5p* can inhibit the expression of PD-L1 and regulate the growth of pancreatic cancer. As a result, there is an increase in CD4 + and CD8 + T lymphocytes, a decrease in PD-1 + T lymphocytes, and an increase in the production of IFN-γ and TNF-α. It suggests that overexpression of *miR-142-5p* can boost the immune response against tumors by blocking the immune checkpoint pathway, with a specific focus on targeting PD-L1 [139].

It was observed that *LINC00473* acted as a sponge for *miR-195-5p*, which led to an increase in the expression of PD-L1. In cell lines and cancerous tissues of pancreatic cancer, it was noted that the levels of *LINC00473* and PD-L1 increased while *miR-195-5p* decreased. It was found that when *LINC00473* was silenced or *miR-195-5p* was increased, the expression of IFN-γ and IL-4 was elevated, and the expression of MMP-2, MMP-9, and IL-10 was reduced. This resulted in increased apoptosis, as well as inhibition of proliferation, invasion, and migration of pancreatic cancer cells. Furthermore, silencing *LINC00473* or elevating *miR-195-5p* activated CD8 + T cells [140].

When pancreatic cancer cells were exposed to hypoxia treatment, the levels of *miR-519* decreased. However, introducing *miR-519* mimics into these cells resulted in the inhibition of cell invasiveness and the induction of apoptosis, especially under hypoxic conditions. Further research showed that *miR-519* directly targets PD-L1. The expression of PD-L1 rescued the reduced tumorigenic effects caused by the *miR-519* mimic in pancreatic cancer cells under hypoxic conditions [141].

The regulatory axis involving *hsa_circ_0046523/miR-148a-3p*/PD-L1 plays a crucial role in mediating the immunosuppressive microenvironment in pancreatic cancer. Specifically, *hsa_circ_0046523* functions by binding to *miR-148a-3p*, resulting in the upregulation of PD-L1 expression in pancreatic cancer (p values < 0.05)[142].

##### Esophageal cancer

The regulation of circ-VIM, *miR-124-3p*, and PD-L1 in esophageal cancer was investigated by the researchers using bioinformatic tools, luciferase assay, and RNA immunoprecipitation. The results revealed that circ-VIM upregulated *miR-124-3p* and downregulated PD-L1 expression. Additionally, sevoflurane treatment independently upregulated *miR-124-3p*, leading to decreased PD-L1 expression. The inhibition of circ-VIM expression and the application of sevoflurane demonstrated significant effects in suppressing immune escape, as well as multiple oncogenic activities in esophageal cancer. Moreover, these interventions effectively suppressed tumor growth and lung metastases [143]. According to Cheng et al. investigation, both *BLACAT1* and PD-L1 are found at higher levels in tumor tissues and cell lines of esophageal cancer. Also, *BLACAT1* promotes the growth, expansion, and metastasis of esophageal cancer cells by competing with PD-L1 to bind to *miR-5590-3p* in these cells. Moreover, the study found that silencing PD-L1 partially reversed the promoting effects of *BLACAT1* [144].

Table 2 provides an overview of the miRNA-mediated regulation of PD-L1 in gastrointestinal cancers, specifically focusing on colorectal cancer, gastric cancer, pancreatic cancer, and esophageal cancer. It summarizes the different miRNAs involved, their target (PD-L1 or other associated molecules), the mechanisms of action, and the effects on cancer progression and immune response. Table 2 also highlights the intricate regulatory network involving miRNAs and PD-L1, shedding light on potential therapeutic targets and strategies for immunotherapy in gastrointestinal cancers.

**Table 2:** miRNA-mediated regulation of PD-L1, mechanisms of action, and associated effects in gastrointestinal cancers

Gastrointestinal cancer	miRNA targeted PD-L1	Mechanism of action	Effects
Colorectal cancer	*miR-214*	Axis *circEIF3K/miR-214*/PD-L1; Involvement of cancer-associated fibroblasts (CAFs)	Facilitation of colorectal cancer advancement under hypoxic conditions through cancer-associated fibroblasts
	*miR-140-3p*	Direct targeting of PD-L1; Inactivation of the PI3K/AKT pathway	Alleviation of inhibitory effects in tumor cells
	*miR-30a-5p*	*miR-30a-5p/USP22* pathway; Control of PD-L1 ubiquitination	Facilitation of immune evasion
	*miR-15b-5p*	Downregulates PD-L1 expression	Inhibits tumor formation and increases sensitivity to anti-PD-1 treatment
	*miR-21-5p, miR-200a*	Promotes M2-like polarization of TAMs and enhances PD-L1 expression	Decreases T cell activity, enhances tumor growth; potential target for sensitizing CRC to anti-PD-L1 therapy
	*miR-124*	Decreases PD-L1 expression	Inhibits Tregs, reduces cell proliferation, and suppresses tumor growth
	*miR-548b-5p, miR-548c-5p*	Upregulates PD-L1 expression	Promotes tumor growth and metastasis
	*miR-148a-3p*	Downregulates PD-L1 expression	reduces T-cell apoptosis, and decreases PD-L1 levels induced by IFNγ
	*miR-497*	Downregulates PD-L1 expression	Inhibits Tregs, suppresses cell proliferation, migration, and invasion
	*miR-191-5p, miR-382-3p, miR-200a-3p, miR-200c-3p, miR-138-5p*	Suppresses PD-L1 expression	Reduces tumor migration, promotes anti-tumoral immune responses, inhibits tumor growth, decreases cell viability, enhances sensitivity to chemotherapy
Gastric cancer	*miR-105-5p*	Binding to a specific cis-acting region; Negative correlation with PD-L1 expression	Activation of CD8 + T cells
	*miR-940*	Promotion of *STAT5A* ubiquitination; Inhibition of PD-L1 expression	Inhibition of aggressive characteristics
	*miR-429*	Direct targeting of PD-L1; Indirect regulation through *lncRNA HIF1A-AS2*	Inhibition of tumor development
	*miR-502-5p*	Transcriptional and post-transcriptional downregulation of PD-L1	Inhibition of tumor growth and metastasis
	*miR-21*	Upregulation of *miR-21*; Mitigation of Th17/Treg cell imbalance	Reduction of Th17 cell population; Increase in Treg cell population
	*miR-375-3p*	Axis *hsa_circ_0136666/miR-375-3p/PRKDC* and axis *miR-375-3p/JAK2/STAT3*/PD-L1	Upregulation of PRKDC, modulation of immune checkpoint proteins, phosphorylation of PD-L1, immune evasion,
	*miR-16-5p*	Reduction of PD-L1 expression	Induction of T cell immune response, inhibition of tumor formation
	*miR-744-5p*	Regulates PD-L1 expression	Regulation of *SLC7A5*, immune evasion and resistance to anti-PD-1 treatment
	*miR-5193, miR-4443, miR-520 h, miR-496*	Direct targeting of PD-L1	Reduced PD-L1 expression, enhanced anti-tumor activity of T cells
Pancreatic cancer	*miR-382-3p*	*PSMB8-AS1/miR-382-3p/STAT1*/PD-L1 axis; Upregulation of PD-L1 expression	Therapeutic target
	*miR-194-5p*	Negative correlation with PD-L1 expression; Inhibition of migration, invasion, and proliferation	Inhibition of cancer progression; Facilitation of CD8 + T cell infiltration
	*miR-148a-3p*	*hsa_circ_0046523/miR-148a-3p*/PD-L1 axis; Upregulation of PD-L1 expression	Mediation of immunosuppressive microenvironment
	*miR-519*	Direct targeting of PD-L1	Inhibits cell invasiveness and induces apoptosis under hypoxic conditions
	*miR-142-5p*	Inhibition of PD-L1 expression	Increased CD4 + and CD8 + T lymphocytes, reduced PD-1 + T lymphocytes, increased IFN-γ and TNF-α production
	i	Increased PD-L1 expression	Decreased apoptosis, proliferation, invasion, and migration of pancreatic cancer cells
Esophageal cancer	miR-124-3p	Regulation by circ-VIM; Downregulation of PD-L1 expression	Suppression of immune escape and oncogenic activities; Inhibition of tumor growth and metastases
	miR-5590-3p	Competitive binding with PD-L1	Promoting growth, expansion, and metastasis of tumor cells, partially reversed by silencing PD-L1

#### miRNA-mediated regulation of PD-L1 in hepatocellular carcinoma (HCC)

In HCC cells, the EGFR-P38 MAPK pathway has the ability to increase the expression of PD-L1 by utilizing *miR-675-5p*. There was a positive correlation between phosphorylated EGFR (p-EGFR) and PD-L1 expression. Activation of EGFR by its ligand EGF resulted in the upregulation of PD-L1 in HCC cells. Additionally, the downregulation of *miR-675-5p* led to increased stability of PD-L1 mRNA, likely through the 3'-UTR of PD-L1, resulting in the accumulation of PD-L1 [145].

The presence of endoplasmic reticulum (ER) stress in HCC cells triggers the release of exosomes. These exosomes have the ability to dampen the antitumor immune response by affecting the expression of PD-L1 in macrophages. Specifically, ER-stressed HCC cells release exosomes that enhance the expression of PD-L1 in macrophages. This, in turn, hampers the function of T-cells through an exosome-mediated pathway involving *miR-23a*, *PTEN*, and *AKT* [146]. *miR-145* directly targeted PD-L1, and *hsa_circ_0003288* functioned as a sponge for *miR-145*, modulating PD-L1 expression via the PI3K/Akt pathway. Elevated levels of *hsa_circ_0003288* resulted in increased PD-L1 expression and facilitated EMT, as well as enhanced migration and invasion of HCC cells [147]. Furthermore, *LINC00657* regulated PD-L1 expression by sponging *miR-424*, thus affecting the development of HCC [148].

hsa_circ_0005239 interacts with miR-34a-5p and acts as a ceRNA to regulate the expression of PD-L1. More experiments demonstrated that the *hsa_circ_0005239*/PD-L1 axis plays a regulatory role in the malignant characteristics of HCC cells via the phosphoinositide-3 kinase/protein kinase B (PI3K/Akt) signaling pathway (p values < 0.05) [149].

The MIAT/*miR-411-5p*/STAT3/PD-L1 pathway is recognized as a potential therapeutic target for HCC. In HCC tissues, both MIAT and PD-L1 exhibit significant upregulation, and the expression of PD-L1 is regulated by MIAT. MIAT downregulates *miR-411-5p*, which in turn upregulates STAT3, ultimately leading to increased transcriptional expression of PD-L1 (p values < 0.05) [150].

*HOXA-AS3*, by acting as a sponge for *miR-455-5p*, upregulated the expression of PD-L1 in HCC. Moreover, the effects on cell proliferation and invasion caused by the overexpression of *HOXA-AS3* were reversed when PD-L1 was inhibited and *miR-455-5p* was overexpressed. In summary, *HOXA-AS3* regulated the activities of HCC cells through the *miR-455-5p*/PD-L1 axis [151].

#### miRNA-mediated regulation of PD-L1 in glioblastoma and glioma

The importance of *miR-10b-5p*-mediated suppression of Ten-eleven translocation 2 (TET2) in immune evasion induced by PD-L1 and suggests their potential as targets for immunotherapy in glioblastoma (GBM) are demonstrated. Suppression of *miR-10b-5p* weakens the survival, migration, invasion, release of immunosuppressive factors, T cell apoptosis, and cytotoxicity in GBM cell models. This suppression regulates TET2, which is downregulated in GBM, impacting the aggressiveness of GBM cells and their ability to evade the immune system. TET2 recruits HDAC1 and HDAC2 to the PD-L1 promoter, leading to the inhibition of PD-L1 transcription [152].

The inhibition of PD-L1 has shown promise in enhancing the effectiveness of radiotherapy by suppressing DNA damage and repair responses. In the case of GBM, the *miR-33a-5p* plays a critical role in promoting tumor growth and self-renewal. To explore the potential of using a PD-L1 inhibitor to modulate *miR-33a-5p* and exert anti-tumor effects in GBM cells, researchers conducted an investigation. They discovered that the PD-L1 inhibitor increased the sensitivity of GBM cells to radiotherapy. This effect was achieved by inhibiting *miR-33a-5p*, which resulted in the activation of PTEN and the induction of DNA damage. These findings highlight the importance of targeting *miR-33a-5p* and inducing DNA damage for effective antitumor immunotherapies in the treatment of GBM (p values < 0.05) [153].

Additionally, the study discovered a strong association between the concentration of *miR-155* and the expression level of PD-L1 in tumor tissue of high-grade pediatric glioma (p-HGG) [154].

#### miRNA-mediated regulation of PD-L1 in neuroblastoma, head and neck squamous cell carcinoma and thyroid carcinoma

In neuroblastoma (NB), *miR-15a* and *miR-15b* trigger an immune response against tumors by specifically targeting PD-L1. PD-L1 plays a crucial role in immune evasion, but by targeting PD-L1 with *miR-15a* and *miR-15b* oligonucleotides, immune cell activation, cytokine secretion, NB cell cytotoxicity, and an anti-tumor immune response can be induced [155].

The *circ_0000052/miR-382-3p*/PD-L1 axis is of crucial significance in the progression of head and neck squamous cell carcinoma (HNSCC). Higher levels of PD-L1 are associated with HNSCC recurrences and play a pivotal role in regulating various cellular processes, including proliferation, migration, invasion, clonogenicity, and apoptosis. The study not only confirms that the IFN-γ/JAK2/STAT1 signaling pathway can induce PD-L1 overexpression in HNSCC but also uncovers a novel mechanism by which the upregulated *circ_0000052* mediates PD-L1 overexpression. Specifically, *circ_0000052* competes with miR-382-3p for binding and alleviates its inhibitory effect on PD-L1 expression (156).

In Follicular thyroid carcinoma (FTC), there is an increase in the expression of PD-L1, which is strongly linked to the aggressiveness and advancement of the tumor. Additionally, PD-L1 expression is positively correlated with Claudin-1 expression. Eventually, *MiR-199a-5p* plays a functional role in the progression and metastasis of FTC by regulating the expression of PD-L1 and Claudin-1 (p values < 0.05) [157].

In anaplastic thyroid carcinoma (ATC), both UCA1 and PD-L1 exhibit elevated expression levels. UCA1 acts as a negative regulator of *miR-148a*, which in turn targets PD-L1 to reduce its expression. Moreover, UCA1 hinders the cytotoxic effects of CD8 + T cells and reduces cytokine secretion by means of the PD-L1 and *miR-148a* pathways [158].

#### miRNA-mediated regulation of PD-L1 in breast cancers

*miR-4759* was identified as a suppressor of PD-L1 gene expression. It targeted the PD-L1 gene by binding to two specific regions in its 3' untranslated region (3'-UTR). Restoring *miR-4759* expression resulted in a reduction of PD-L1 levels and increased susceptibility of breast cancer cells to immune cell-mediated killing. In vivo studies using immunocompetent mice demonstrated that treatment with *miR-4759* suppressed tumor growth and promoted infiltration of CD8 + T lymphocytes into the tumor. However, *miR-4759* did not affect tumor growth in immunodeficient mice(p values < 0.05) [159].

The overexpression of Tissue differentiation-inducing non-protein coding RNA (TINCR) led to increased PD-L1 expression and facilitated the progression of breast cancer. Conversely, when TINCR was suppressed, the therapeutic efficacy of PD-L1 inhibitors in breast cancer was significantly improved. The underlying mechanism involves TINCR recruiting DNMT1 to induce methylation of the *miR-199a-5p* gene, suppressing its transcription. Additionally, in the cytoplasm, TINCR potentially functions as a molecular sponge for *miR-199a-5p*, thereby promoting PD-L1 expression by reducing its ubiquitination levels [160]. The levels of *miR-355* and *miR-145* were markedly reduced in breast cancer tissues, and this reduction exhibited a negative association with the overexpression of PD-L1 [161].

Exosomes carrying *miR-27a-3p* were found to enhance immune evasion in breast cancer by increasing the expression of PD-L1 through the MAGI2/PTEN/PI3K axis. Breast cancer tissues exhibited higher levels of ER stress biomarkers, including PD-L1, compared to adjacent non-cancerous tissues. Additionally, macrophages exposed to exosomes containing elevated levels of *miR-27a-3p* showed increased expression of both *miR-27a-3p* and PD-L1. The *miR-27a-3p* molecule was capable of targeting and negatively regulating MAGI2, while MAGI2, in turn, down-regulated PD-L1 levels by up-regulating PTEN to deactivate the PI3K/AKT signaling pathway [162]. As well, *miR-93-5p* plays a role in regulating both tumorigenesis and tumor immunity by targeting PD-L1/CCND1 in breast cancer. By directly targeting the PD-L1/CCND1 signaling pathway, *miR-93-5p* affects the accumulation and distribution of these molecules in various cellular compartments, including the cell membrane, nucleus, and cytoplasm. This regulation ultimately influences tumor progression and immune responses (p values < 0.05)[163].

Moreover, the findings indicated that *miR-34a* can suppress the growth of tumors and reduce the levels of PD-L1 in TNBC [164].

#### miRNA-mediated regulation of PD-L1 in cervical, ovarian, and endometrial cancer

Patients diagnosed with cervical cancer caused by human papillomavirus (HPV) and who test positive for it, exhibit an imbalance in the normal vaginal microbial composition, also known as dysbiosis. Additionally, these patients have elevated levels of *miR-18a* and PD-L1 in their bloodstream. Notably, patients in stage III of the disease present higher levels of *miR-18a* and PD-L1 compared to those in stages I and II [165]. The presence of HPV in cervical cancer cells may facilitate immune evasion by modulating the *miR-142-5p*/PD-L1 axis (p values < 0.05) [166].

A research study revealed that overexpression of *EMX2OS* contributes to increased proliferation, invasion, and sphere formation of ovarian cancer. *EMX2OS* directly binds to *miR-654*, leading to the suppression of miR-654 expression. Consequently, the expression of AKT3, a target of *miR-654*, is upregulated. Additionally, it is noteworthy that PD-L1 is identified as a crucial oncogenic component in ovarian cancer cells and functions downstream of AKT3. The reversal of anti-cancer functions caused by *EMX2OS* knockdown, AKT3 silencing, or miR-654 upregulation can be restored by ectopic expression of PD-L1 [167]. The *FGD5-AS1/miR-142-5p*/PD-L1 axis is implicated in the regulation of ovarian cancer progression. FGD5-AS1 specifically targets *miR-142-5p*, leading to its downregulation and diminished functionality. Additionally, *miR-142-5p* has a binding site within the 3' UTR of PD-L1, and *FGD5-AS1* positively modulates PD-L1 expression by suppressing *miR-142-5p* [168].

It is noteworthy, *miR-216a* directly targets PD-L1 and plays a role in regulating PD-L1 levels in aggressive endometrial cancer cells. Additionally, it reveals that MEG3 and *miR-216a* act as upstream regulators, influencing the expression of PD-L1 (p values < 0.05) [169].

#### miRNA-mediated regulation of PD-L1 in diffuse large B cell lymphoma and melanoma

By targeting PD-L1, *miR-214* regulates the progression of Diffuse large B cell lymphoma (DLBCL). Overexpression of miR-214 inhibited tumor growth by targeting PD-L1 in vivo [170]. There was an inverse association between *miR-195* and both *MALAT1* and PD-L1. *MALAT1* acted as a sponge for *miR-195*, thereby controlling the expression of PD-L1. In conclusion, *MALAT1* functioned as a regulator of proliferation, apoptosis, migration, and immune evasion in DLBCL by sequestering miR-195 and modulating the expression of PD-L1 [171].

In melanoma cells, there is an elevated expression of *KCNQ1OT1*, which affects the *miR-34a/STAT3* pathway, leading to the enhancement of expansion, migration, and aggression capabilities. Likewise, *KCNQ1OT1* hinders the function of CD8 + T cells through the *miR-34a/STAT3*/PD-L1 axis, thereby facilitating immune evasion by melanoma cells [172].

#### miRNA-mediated regulation of PD-L1 in bladder and prostate cancer

Autophagy-related gene 7 (*ATG7*) plays a role in regulating PD-L1 protein in bladder cancer. Increased expression of *ATG7* leads to elevated levels of PD-L1 protein by promoting autophagy-dependent degradation of FOXO3a, which in turn inhibits the transcription of *miR-14*5, a molecule known to regulate PD-L1 expression. Consequently, this downregulates the expression of *miR-145*, resulting in enhanced stability and expression of PD-L1 mRNA. This, in turn, promotes the acquisition of stem-like properties and increased invasion capabilities in bladder cancer cells. The deficiency in autophagy activation, FOXO3A degradation, and *miR-145* transcription attenuation observed in ATG7 knockdown cells is reversed by the overexpression of PD-L1 (p values < 0.05) [173].

In prostate cancer, *miR-15a* directly interacts with the 3'-untranslated region (UTR) of PD-L1, leading to the inhibition of PD-L1 expression. The overexpression of *miR-15a* in prostate cancer cells alone is adequate to enhance cytotoxicity and proliferation. Additionally, *KCNQ1OT1* acts as a sponge for *miR-15a*, facilitating immune evasion and promoting the malignant progression of prostate cancer by upregulating PD-L1 [174].

### microRNA regulation of CTLA-4 in cancer

CTLA-4, an immune checkpoint present in regulatory T (Treg) cells and activated T lymphocytes, has limited effectiveness when inhibited for cancer treatment. In a study focused on metastatic melanoma, it was discovered that decreased CTLA-4 mRNA levels were associated with a worse prognosis. The researchers identified Treg cells as the source of this downregulation of CTLA-4 in metastatic melanoma patients. They further observed that secretomes from human metastatic melanoma cells suppressed CTLA-4 mRNA through* miR-155* while simultaneously increasing *FOXP3* expression in human Treg cells. The expression of CTLA-4 inhibited the proliferation and suppressive function of human Treg cells. Consequently, targeting *miRNA-155* or other factors involved in CTLA-4 regulation specifically in Treg cells, without affecting T cells, has the potential to enhance the effectiveness of immunotherapy in treating melanoma [175].

### microRNA regulation of PD-1 and PD-L1 in cancer

The research demonstrates that lncNDEPD1 can interact with *miR-3619-5p* and PDCD1 mRNA in CD8 + T Cells, thereby preventing mRNA degradation and increasing the expression of PD-1. Furthermore, Notch1 directly binds to the promoter region of *lncNDEPD1*. In chimeric antigen receptor (CAR) T cells expressing *lncNDEPD1*-specific RNAs, the presence of PD-L1 enhances their tumor-killing abilities [176].

There is a strong correlation between *miR-223* expression and tumor hypoxia, which is crucial for the regulation of PD-1/PD-L1. *MiR-223* indirectly suppressed PD-1 and PD-L1 expression in immune cells, modulating the tumor microenvironment. It also hindered angiogenesis and inhibited hypoxia-induced activation of PD-1/PD-L1 in HCC models [177].

In the tumor microenvironment, *let-7b* has been found to post-transcriptionally inhibit the expression of PD-L1 and PD-1. This suggests that *let-7b* miRNAs may have a role in enhancing the body's immune response against tumors in vivo. The treatment has the potential to reduce PD-1 expression in both CD8 + T cells and lung tumor cells, which may contribute to the prevention of lung cancer [178]. Furthermore, exosomal *miR-16-5p* has the potential to act as a latent inhibitor of tumor growth and serve as a biomarker for PD-L1 inhibitor-dependent immunotherapy in lung adenocarcinoma. This is achieved through the regulation of PD-L1 expression (179).

Zhang et al. investigated the potential of aerosolized miRNA mimics, specifically *miR-138-5p* and *miR-200c*, for lung cancer prevention. The combined administration of these miRNA mimics successfully inhibited the development of Benzo(a)pyrene-induced lung adenomas and N-nitroso-tris-chloroethylurea-induced lung squamous cell carcinomas. The miRNAs were additionally effective in suppressing the expression of PD-L1. The administration of the combined miRNAs increased in CD4 + and CD8 + T cells, while reducing the expression of PD-1 and T-regulatory cells. These findings suggest that aerosolized miRNAs specifically targeting PD-L1 can be an effective approach in preventing the development and progression of lung cancer in mice (p values < 0.05) [180].

The researchers discovered that *miR-346, miR-328-3p, miR-326, and miR-330-5p* bind to CD155 mRNA. This interaction is considered to be one of the ways through which resistance to PD-1 and PD-L1 inhibitors occurs. Consequently, *miR-326* acts as a negative regulator of CD155 expression in lung adenocarcinoma, and as a result, it may have a role in the emergence of resistance to PD-1/PD-L1 inhibitors [181].

In a mouse model, thalidomide demonstrated the ability to decrease tumor growth and angiogenesis while increasing the ratio of CD8 + T cells. Its target was *FGD5-AS1*, a gene associated with unfavorable prognosis in patients with NSCLC. *FGD5-AS1* functioned as a *miR-454-3p* sponge, resulting in the upregulation of *ZEB1*. This upregulation, in turn, led to increased expression of PD-L1 and *VEGFA* while simultaneously inhibiting cancer cell proliferation and apoptosis. Thalidomide suppressed angiogenesis and immune evasion in NSCLC by modulating the FGD5-AS1/miR-454-3p/ZEB1 axis. This regulatory pathway influenced the expression of *VEGFA* and the PD-1/PD-L1 checkpoint, contributing to the inhibitory effects of thalidomide [182]. Besides, exosomal *miR-125a-3p* holds promise as a potential indicator of the likelihood of positive response to anti-PD-1/PD-L1 therapy in advanced NSCLCs that have low PD-L1 expression levels. Elevated levels of *miR-125a-3p* were linked to poorer progression-free and overall survival rates. In NSCLC cells, the introduction of *miR-125a-3p* resulted in the regulation of PD-L1 expression by suppressing neuregulin 1 (NRG1) (p values < 0.05) [183].

The expression levels of exosomal *miRNA-146a* and *miRNA-126*, which are associated with the regulation of immune responses, were examined in clear cell renal cell carcinoma (ccRCC) patients before and after PD-1 and PD-L1 therapy [184].

*miR-146a* has a key role in the STAT1/IFNγ axis within the melanoma microenvironment, influencing melanoma cell migration, proliferation, mitochondrial function, and PD-L1 expression. Furthermore, the simultaneous inhibition of PD-1 and miR-146a may represent a promising approach to augment the antitumor immune response triggered by checkpoint therapy [185].

The miR*-29-5P/ARID3A* axis forms a negative feedback loop that regulates the expression of PD-L1 in DLBCL, influencing the activity of CD8 + T cells and facilitating the immune evasion of tumor cells. Inhibition of *miR-129-5p* and the overexpression of *ARID3A* in lymphoma cells were found to enhance immune escape by upregulating PD-L1 expression, which was under the transcriptional control of ARID3A. In summary, the *miR-129-5P/ARID3A* negative feedback loop plays a role in modulating DLBCL progression and immune evasion by regulating the PD-1/PD-L1 pathway (p values < 0.05) [186].

The use of naturalizing PD-1 and PD-L1 to inhibit the immune checkpoint has been demonstrated as an effective treatment strategy for lymphoma. small nucleolar RNA host gene 14 (*SNHG14*) was found to enhance the proliferation, invasion, EMT, and tumor growth of Diffuse large B cell lymphoma (DLBCL). Besides, a positive feedback loop was identified between *SNHG14*, *miR-5590-3p*, and ZEB, which resulted in the activation of PD-L1. This activation of PD-L1 led to the inactivation of CD8 + T cells, ultimately aiding in the immune evasion of DLBCL cells. Therefore, targeting *SNHG14* could hold promise as an immunotherapeutic approach by blocking the PD-L1/PD-1 pathway in DLBCL [187].

In DLBCL, the expression of the *SNHG14*is elevated, while the expression of miR-152-3p is reduced. *SNHG14* promotes the growth, migration, and epithelial-mesenchymal transition (EMT)-like processes of DLBCL cells in vitro. It also suppresses the expression of *miR-152-3p*. The *SNHG14/miR-152-3p* axis inhibits apoptosis and enhances cell proliferation by affecting cytotoxic T lymphocytes (CTLs) in DLBCL through modulation of the PD-1/PD-L1 immune checkpoint [188].

The targeting of *miR-146b* can be utilized as a complement to anti-PD-1 immunotherapy in colorectal cancer. Deletion of *miR-146b* resulted in the accelerated advancement of tumors, primarily due to an increase in the population of alternatively activated (M2) tumor-associated macrophages. This process was regulated by m6A-related proteins, METTL3 and *HNRNPA2B1*, which influenced the maturation of *miR-146b*. Consequently, the polarization of M2-TAMs was promoted, and the PI3K/AKT signaling pathway was enhanced, contributing to tumor progression. The absence of miR-146b led to a decrease in T cell infiltration, exacerbating immunosuppression and facilitating tumor advancement. Furthermore, the deletion of *miR-146b* stimulated the production of PD-L1 in TAMs, thereby enhancing the efficacy of anti-PD-1 immunotherapy against the tumor. Consequently, targeting *miR-146b* has the potential to act as a supplementary approach to augment anti-PD-1 immunotherapy (p values < 0.05) [189].

The *lncRNA SNHG4* acts as a sponge for *miR-144-3p*, leading to the upregulation of c-MET. This upregulation of c-MET promotes cell proliferation, colony formation, invasion, and immune evasion. Furthermore, overexpression of *SNHG4* or knockdown of *miR-144-3p* activated PD-1/PD-L1 and induced CD4 + T cell apoptosis in colorectal cancer [190].

The *miR-BART5*-5p molecule directly targets PIAS3 and enhances the expression of PD-L1 through the regulation of the *miR-BART5/PIAS3/pSTAT3*/PD-L1 axis in EBV-associated gastric carcinomas. This mechanism contributes to anti-apoptosis, increased tumor cell proliferation, invasion, migration, and immune evasion, ultimately promoting the progression of gastric carcinoma and worsening clinical outcomes. Considering its involvement in PD-L1 regulation, *miR-BART5-5p* may represent a potential target for PD-1/PD-L1 immune checkpoint inhibitor therapy [191].

### microRNA regulation of PD-1, PD-L1, and CTLA-4 in cancer

High levels of *miR-33a-5p* were detected in lung adenocarcinoma, which was linked to reduced expression of PD-1, PD-L1, and CTLA-4. Additionally, the elevated expression of *miR-33a-5p* was associated with a more favorable prognosis [192].

*miR-424* has been identified as a regulator that targets both the CTLA-4/CD80 and PD-1/PD-L1 pathways. Also, a significant correlation between *miR-424-3p* and CTLA-4 was confirmed [193].

### Methodology for identifying miRNAs regulating PD-1 and PD-L1 expression

One of the most crucial techniques in identifying miRNAs that regulate ADs involves bioinformatic analysis. The first step is to conduct comprehensive bioinformatics analyses that use databases and prediction algorithms such as TargetScan, miRanda, PITA, SVmicrO, RNA22, picTar and miRBase. Through these analyses, potential miRNA candidates that could target the 3' untranslated regions (UTRs) of PD-1 and PD-L1 mRNA are identified [115, 194]. Once the bioinformatics analysis is complete, experimental validation is conducted to confirm the regulatory effects of the identified miRNAs on PD-1 and PD-L1 expression. This involves the use of animal models, human tissues, and cell culture models such as cancer cell lines or primary cells that express PD-1 and PD-L1. The specific miRNA mimics or inhibitors are transfected into these cells to modulate the expression of the selected miRNAs. Appropriate controls, including negative control miRNAs or mock transfections, are included to account for non-specific effects [177].

The expression levels of PD-1 and PD-L1 are assessed using techniques such as quantitative real-time polymerase chain reaction (qRT-PCR), Western blotting and Immunohistochemical analyses (IHC). These analyses allow for the measurement of PD-1 and PD-L1 mRNA levels and protein expression, respectively. Other techniques included NanoString nCounter® assay and ELISA assays [88, 121, 192, 195, 196]. Both the transfected cells and control groups are analyzed to compare the changes in PD-1 and PD-L1 expression upon modulation of the specific miRNAs. To validate the direct interaction between the identified miRNAs and the 3' UTRs of PD-1 and PD-L1 mRNA, luciferase reporter assays are performed. The 3' UTRs of PD-1 and PD-L1 are cloned downstream of a luciferase reporter gene, and these constructs are co-transfected with the specific miRNA mimics or inhibitors. Luciferase activity is measured to determine whether the selected miRNAs directly target and regulate the 3' UTRs of PD-1 and PD-L1 [115] [83, 121, 197].

## The role of extracellular vesicles and miRNAs in tumor progression and immune modulation

It is widely recognized that both cancer cells and cells within the tumor microenvironment release EVs, including exosomes and microvesicles (MVs). These EVs can be detected in various biological fluids of the body, such as blood, urine, sperm, and others. Exosomes, a subtype of small membrane vesicles, play a role in facilitating tumor progression by transporting proteins, bioactive substances, mRNAs, miRNAs, and other crucial agents required for the essential functions of cancer cells [198]. EVs released by tumor cells have been observed to inhibit the immune system response, deactivate T lymphocytes and natural killer cells, and facilitate the differentiation of regulatory T lymphocytes, thereby promoting tumor growth. In overview, miRNAs have emerged as key regulators in shaping cancer and their involvement in modulating innate and adaptive immune responses through the regulation of key factors in immune checkpoints has been established [199, 200].

The initial level of *miR-625-5p* associated with EVs was found to be linked to survival outcomes in NSCLC patients treated with immune checkpoint inhibitors. Interestingly, despite the correlation between *miR-625-5p* expression and high PD-L1 levels, it was able to identify patients who did not respond to ICI treatment, even when over-expression of PD-L1 [201].

In another way, urinary EVs that carry *miR-224*-5p have been identified as a potential biomarker indicating invasive and metastatic capabilities in renal cell carcinoma (RCC). The role of *miR-224-5p* in RCC progression has been elucidated through its ability to regulate PD-L1 protein expression by suppressing CCND1. This discovery sheds light on new functions of *miR-224-5p* and its involvement in RCC progression [202].

Notably, EVs have the potential to function as a delivery platform for innovative immunotherapies targeting TNBC by utilizing the *miR-424-5p*/PD-L1 pathway. Zhou et al. demonstrated that the delivery of *miR-424-5p* using EVs derived from adipose tissue-mesenchymal stromal cells (AT-MSCs) contributes to the promotion of proinflammatory responses and enhances cytotoxicity against tumors. Additionally, High levels of PD-L1 and a positive correlation between PD-L1 expression and overall patient survival have been found in TNBC, also, this expression could be decreased by *miR-424-5p* regulation [203].

**Fig. 3:**
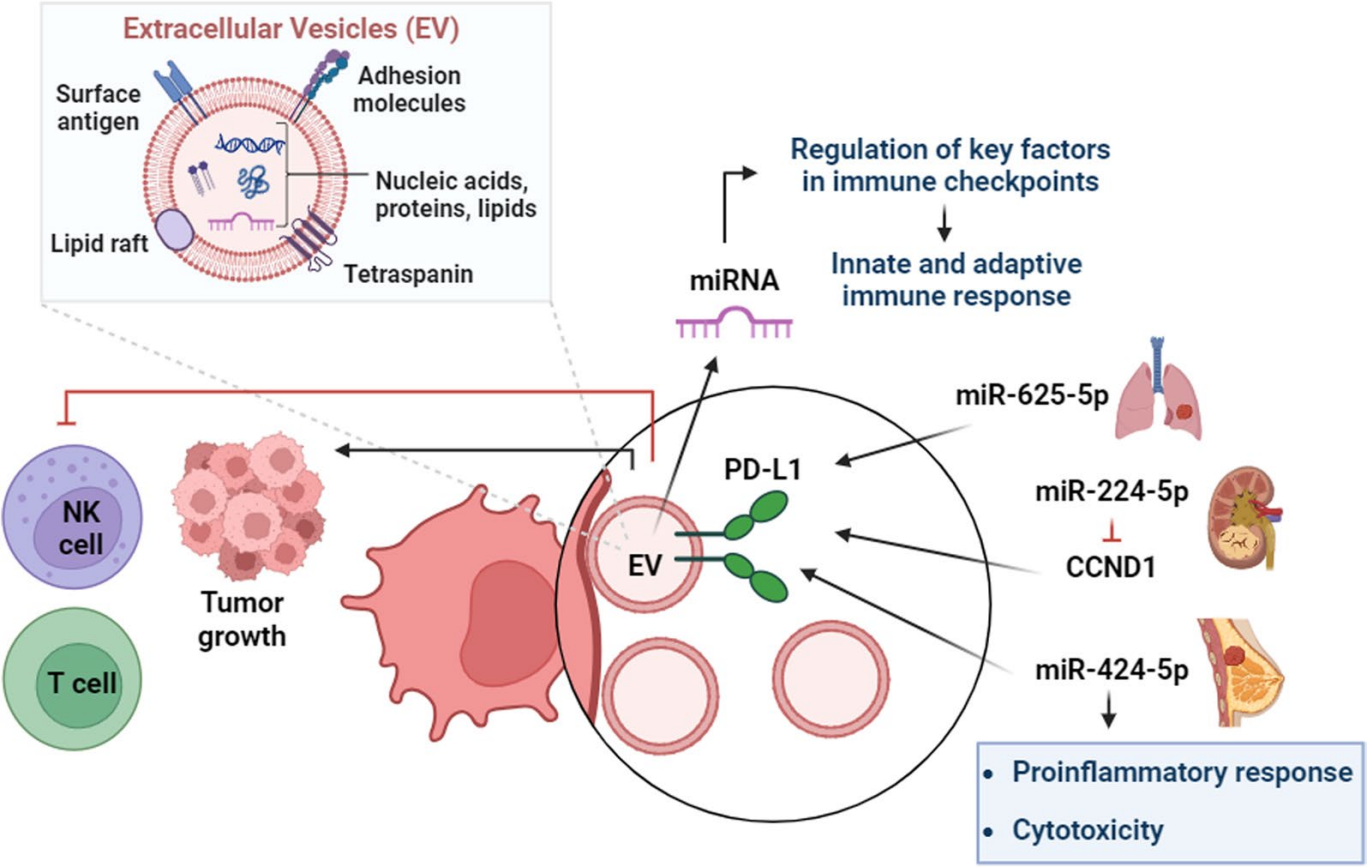
The interplay between extracellular vesicles (EVs), miRNAs, tumor progression, and immune modulation in cancer

Accordingly, the Fig. 3 illustrates the interplay between extracellular EVs, miRNAs, and immune modulation in cancer. It highlights the functions of EVs in cancer progression, immune system inhibition, and the role of specific miRNAs in regulating key factors in immune checkpoints.

## miRNA-mediated regulation anti-PD-1 and anti-PD-L1 treatment: implications for chemoresistance and immunotherapy

Chemoresistance, which refers to the resistance of cancer cells to chemotherapy drugs, poses a significant challenge in cancer treatment. In this context, PD-1 and PD-L1, proteins involved in immune regulation, have been implicated in chemoresistance and are found to be overexpressed in various types of human cancer cells.

The miR-873/PD-L1 regulatory axis shows potential as a therapeutic target for improving the responsiveness to chemotherapy and eliminating the stem-like properties of breast cancer cells. PD-L1 expression is linked to stemness markers and contributes to chemoresistance and stemness-like properties in breast cancer cells. *MiR-873* inhibits PD-L1 expression by binding to its 3′-UTR, attenuating stemness and chemoresistance. Recombinant PD-1 enhances PD-L1's promotion of stemness and chemoresistance, while PD-1/PD-L1 inhibitors attenuate this effect [204].

### Cisplatin

Cisplatin, an anticancer drug, has been reported to induce the expression of PD-L1, thereby regulating cancer immunity. The role of PD-L1 in regulating resistance to cisplatin is crucial, although the exact mechanisms involved are not completely understood. In a recent study, it was discovered that the expression of *miR-526b-3p* was reduced, while PD-L1 expression was increased in cisplatin-resistant lung cancer samples compared to cisplatin-sensitive lung cancer samples. Importantly, lower levels of *miR-526b-3p* were associated with a poorer response to cisplatin treatment. Additionally, it was shown that *miR-526b-3p* can reverse cisplatin resistance, inhibit metastasis, and activate CD8 + T cells through a mechanism dependent on STAT3/PD-L1 [197]. It has also been found that the increased expression of PD-L1 in acquired cisplatin-resistant lung cancer cells is dependent on *mir-181a* in NSCLC. In NSCLC (A549) cells, the pathway involving cisplatin-induced *ATM-mir-181a-c-FOS* was responsible for suppressing PD-L1 expression. However, in (A549R) cells, this negative regulatory mechanism was blocked, leading to further increased PD-L1 expression [205]. The *circ_0068252/miR-1304-5p*/PD-L1 axis plays a crucial role in regulating both cisplatin resistance and immune evasion in NSCLC cells. Deletion of *circ_0068252* improves the sensitivity of cisplatin-resistant NSCLC cells to cisplatin. Moreover, the suppression of *circ_0068252* has an impact on the immune microenvironment, particularly in relation to CD8 + T cells. Lastly, *circ_0068252* modulates PD-L1 expression by acting as a mediator for* miR-1304-5p* [206]. In cisplatin-resistant ovarian cancer cells, identified an upregulation of PD-L1 expression and a downregulation of *miR-34a-5p*. The inhibition of PD-L1 resulted in a reduction in chemoresistance of cisplatin-resistant ovarian cancer cells to cisplatin. This was demonstrated by decreased cell proliferation, arrest of the cell cycle in the G1 phase, and increased apoptosis. Accordingly, the *miR-34a-5p*/PD-L1 axis plays a role in regulating the chemoresistance of ovarian cancer cells to cisplatin [207]. Cisplatin-resistant ovarian cancer cells exhibit a decrease in the expression of *miR-145*, which is further down-regulated by cisplatin treatment. The reduction of *miR-145* leads to an increase in PD-L1 gene expression. This effect is mediated by the targeting of the c-Myc transcription factor by cisplatin, which subsequently induces apoptosis in T cells. Therefore, the *miR-145*/c-Myc/PD-L1 axis plays a role in promoting cisplatin resistance in ovarian cancer [158].

PD-L1 and cyclin D1 were both targeted by *miR-276-3p* and showed an inverse correlation with the expression of *miR-576-3p*. Furthermore, overexpression of *miR-576-3p* enhanced the sensitivity of ovarian cancer cells to cisplatin by reducing the levels of PD-L1 and cyclin D1. These findings suggest that *miR-576-3p* could be a potential therapeutic target for ovarian cancer treatment [208].

### Bortezomib

CD8 + T cell function is influenced by the involvement of *miR-155*. Notably, when *miR-155* is increased due to the effects of bortezomib, its target genes, suppressor of cytokine signaling 1 (SOCS1) and inositol polyphosphate-5-phosphatase (SHIP1), are downregulated. In the presence of bortezomib, activated CD8 + T cells showed a substantial decrease in PD-1 expression, particularly in the subset of cells that exhibit the SHIP1 + phenotype. These findings highlight a mechanism through which bortezomib induces the downregulation of negative regulatory proteins, specifically SOCS1 and SHIP1, via *miR-155*. This downregulation ultimately results in the inhibition of PD-1-mediated T-cell exhaustion [209].

### Pembrolizumab

Pembrolizumab, previously known as lambrolizumab, is a monoclonal antibody with a high affinity for the PD-1 receptor. It is a humanized IgG4 kappa antibody that specifically targets PD-1 and is used in the treatment of different types of cancers. Notably, Pembrolizumab has been shown to enhance survival in patients with chemotherapy-resistant gastric cancer. Additionally, it has received FDA breakthrough therapy designation for advanced NSCLC and for the therapy of metastatic squamous and non-squamous NSCLC whose tumors express PD-L1 [210–212]. Pembrolizumab is an immune checkpoint inhibitor that suppresses the advancement of tumors by repressing the PD-L1/PD-1 pathway. This highlights the significance of utilizing antibody-based immune blockade therapies to block the PD-1 pathway in the treatment of cancer [213]. The sensitivity of tumor cells to radiation therapy is enhanced by either treatment with pembrolizumab or the overexpression of *miR-20b-5p*. This was achieved by targeting PD-L1 and deactivating the PD-L1/PD1 pathway. Conversely, when *miR-20b-5p* overexpression was combined with pembrolizumab, it further increased the sensitivity of cancer cells to radiation therapy by suppressing the activity of PD-L1 and PD1 [214].

### Nivolumab

The alterations in *miR-200c* and *miR-34a* expression levels are associated with the response and outcome in advanced NSCLC treated with anti-PD1 (Nivolumab) immunotherapy [215].

The decreased levels of *miR-339* and the higher expression of PD-L1 in tumors lead to a weakened immune response against tumors in patients with renal cell carcinoma (RCC). Consequently, the increase in miR-339 expression following nivolumab treatment aligns with the longer progression-free survival (PFS) observed in patients who responded well to the treatment [216].

The evidence provides the association between reduced expression of *miR-320* and *miR-375* and the positive clinical outcomes of nivolumab therapy in patients with advanced NSCLC. The effectiveness of the treatment was supported by observed increases in exoPD-L1 levels and the fraction of PD1 + CD8 + T-cells, along with a decrease in immunosuppressive cytokines. These findings suggest a link between the expression of these miRNAs and the underlying processes involved in the clinical response [217].

Checkpoint inhibitors including ipilimumab (which targets CTLA-4, a protein associated with cytotoxic T lymphocytes) as well as nivolumab and pembrolizumab (which inhibit programmed cell death protein-1 or PD-1), are responsible for revolutionary change in the treatment of metastatic melanoma. Following anti-PD-1 treatment, there is a noticeable rise in *miR-155* expression both within living organisms and in the specific location being studied, along with decreased levels of PTPN2. Additionally, melanoma patients exhibited elevated *miR-155* levels. The decrease in *miR-155* targets after anti-PD-1 treatment correlated with extended overall survival [218, 219] (Table 3).

**Table 3:** Associations between nivolumab and miRNAs in different cancer types

Cancer type	miRNA	Expression levels	Association with nivolumab response
NSCLC	*miR-200c, miR-34a*	Increased	Response and outcome
	*miR-320, miR-375*	Decreased	Positive clinical outcomes, decrease in immunosuppressive cytokines
RCC	*miR-339*	Decreased	Weakened immune response against tumors; progression-free survival (PFS)
Metastatic melanoma	*miR-155*	Increased	Overall survival; decreased levels of *PTPN2*

### Doxorubicin

A study showed that the downregulation of PD-L1 expression in colorectal cancer cells was induced by doxorubicin (DOX) treatment. In contrast, in breast cancer cells, the expression of PD-L1 initially surpassed the maximum level typically observed in cancer cells, but decreased following DOX treatment. In DOX-treated colorectal cancer cells, there was a reduction in the expression of *miR-140*, whereas in DOX-treated breast cancer cells, its expression increased. On the other hand, the expression of *miR-34a* increased in both types of cells following DOX treatment. Furthermore, a negative association between PD-L1 and *miR-140* was identified in colorectal cancer cells treated with DOX [220].

### Rituximab plus cyclophosphamide, doxorubicin, vincristine, and prednisone (R-CHOP)

Following the administration of rituximab plus cyclophosphamide, doxorubicin, vincristine, and prednisone (R-CHOP) chemotherapy, gastric diffuse large B-cell lymphoma (GDLBCL) patients who exhibited negative PD-L1 and positive miR-34a expression experienced an increase in the rate of complete response. The therapeutic potential of targeting the PD-L1 and *miR-34a* pathway through immunotherapies may be significant in the treatment of GDLBCL [221].

The *circPCBP2/miR-33a/b*/PD-L1 pathway is recognized as a potential diagnostic indicator and therapeutic target for DLBCL. In this pathway, *circPCBP2* interacts directly with miR-33a/b, while *miR-33a/b* targets the 3'-UTR of PD-L1. Overall, *circPCBP2* promotes the stemness of DLBCL cells but reduces their responsiveness to CHOP treatment by sequestering *miR-33a/b*, thereby allowing for increased expression of PD-L1 [222]. Furthermore, the decreased expression of *miR-424-5p* leads to enhanced drug resistance in DLBCL cells by modulating the PD-1/PD-L1 signaling pathway. *MiR-424-5p* directly targets PD-L1, and introducing this microRNA through transfection resulted in increased resistance of CRL2631 cells to CHOP drugs. Conversely, transfection of *miR-424-5p* mimics reduced resistance and PD-L1 expression levels. The inhibitory impact of *miR-424-5p* on PD-L1 was reversed by overexpressing PD-L1 [223].

### Polydatin

Polydatin, a main effective component of the Chinese herb Polygonum cuspidatum, has multiple antitumor activities that inhibit CRC cell proliferation and promote apoptosis by regulating *miR-382*/PD-L1 axis. Polydatin could suppress the expression of PD-L1 by upregulating its target *miR-382* [224].

### Paclitaxel

PD-L1 has the ability to boost the expression of miR-21 by increasing the accumulation of STAT3 on the *miR-21* promoter. Additionally, exosomal PD-L1 has been implicated in promoting drug resistance to paclitaxel by regulating the STAT3/miR-21/PTEN/Akt pathway and enhancing the tumorigenic characteristics of esophageal cancer. Combining anti-PD-L1 treatment with chemotherapy has also shown promise in reducing tumor size. Moreover, we have observed the involvement of PD-L1, *miR-21*, and the multidrug resistance (MDR1) gene in the process of exosomal transfer [225].

### Fluorouracil, oxaliplatin, and docetaxel

The concurrent administration of fluorouracil and oxaliplatin was found to effectively inhibit the malignant behavior of colon cancer cells. This combination therapy prominently reduced the expression of PD-L1 through the involvement of the *miR-183-5p/SOCS3* axis [226].

*LINC00184* has been linked to both resistance to docetaxel and unfavorable prognosis in prostate cancer. Similarly, it plays a role in regulating both docetaxel resistance and the immune response mediated by T-cells in prostate cancer cells. Specifically, *LINC00184* triggers the recruitment of *miR-105-5p* and has a negative regulatory effect on it, resulting in the suppression of PD-L1 expression levels [227].

### Mifepristone, cetuximab and carboplatin

Mifepristone can downregulate PD-L1 expression in ovarian cancer cells through the *miR-127-3p/VAMP2* axis, thereby inhibiting cancer progression. Mifepristone treatment leads to an upregulation of *miR-127-3p* expression. Consequently, mifepristone significantly suppresses the proliferation of ovarian cancer cells, promotes apoptosis, and inhibits the expression of PD-L1 [228].

In CRC tissue, increased expression of HCG18 and PD-L1 has been shown, while decreased expression of *miR-20b-5p*. Through functional analysis, it was found that *lncRNA HCG18* facilitates growth, motility and resistance to cetuximab in CRC cells by affecting the *miR-20b-5p*/PD-L1 pathway [229].

Upregulation of *miR-766-5p* in ovarian cancer cells enhanced their responsiveness to carboplatin treatment. It was also confirmed that PD-L1 is targeted by *miR-766-5p*. Additionally, *LINC01503*, increased the levels of PD-L1 by modulating miR-766-5p. Notably, GATA1, a transcription factor, stimulated the expression of *LINC01503*, leading to accelerated resistance to carboplatin in ovarian cancer cells through the *miR-766-5p*/PD-L1 pathway [230].

### Trastuzumab

*miR-1184* is a functional target of *circ_0001598* and PD-L1. In addition, *circ_0001598/miR-1184*/PD-L1 signaling plays a crucial role in the regulation of HER2-positive breast cancer progression and trastuzumab-resistance phonotypes [231].

### Atractylodis macrocephalae rhizoma (PAMR)

Atractylodis Macrocephalae Rhizoma (PAMR), a Chinese herbal medicine known for its anti-tumor properties, was found to inhibit the growth of esophageal carcinoma cells. Additionally, PAMR significantly reduced the expression of PD-L1, a protein associated with immune evasion, while enhancing the expression of *miR-34a*, a tumor-suppressing molecule. These results suggest that the main mechanism by which PAMR inhibits PD-L1 expression is through the induction of *miR-34a* [232].

### Nobiletin

Nobiletin, a naturally occurring flavonoid derived from citrus peel, possesses anti-cancer properties. It effectively inhibits the expression of PD-L1 by targeting the EGFR/JAK2/STAT3 signaling pathway in NSCLC. Nobiletin demonstrates p53-independent suppression of PD-L1 and *miR-197* is identified as a regulator of STAT3 and PD-L1 expression [233].

### Thymoquinone (TQ)

Thymoquinone (TQ) extracted from seeds of Nigella sativa induced apoptosis through upregulating ROS level impairing autophagic flux, and inhibiting the EMT and cell invasion via activating the *miR-877-5p*/PD-L1 axis in bladder carcinoma cells [234].

### Olaparib

In HCC, the effectiveness of immune checkpoint therapy can be enhanced by inhibiting the DNA repair enzyme poly (ADP-ribose) polymerase (PARP) through the *miR-513*/PD-L1 pathway. The combination of the PARP inhibitor olaparib and anti-PD1 therapy proves to be advantageous in treating HCC. Importantly, olaparib, as a PARP inhibitor, can elevate the expression of PD-L1 in HCC cells by suppressing *miR-513* [235].

### Oleuropein and sativan

Oleuropein, a secoiridoid glucoside derived from Olea europaea, has emerged as a novel nutri-epigenetic compound in the field of immune-oncology. It exerts control over the m*iR-194/XIST*/PD-L1 loop in TNBC. Analysis of breast cancer biopsies revealed a significant upregulation of *miR-194* and PD-L1 levels, accompanied by a downregulation of XIST. Thus, treatment with Oleuropein demonstrated anti-carcinogenic effects by reducing *miR-194* and PD-L1 expression while increasing *XIST* levels [236]. Furthermore, In TNBC, Sativan (SA), an isoflavane compound derived from natural sources, can suppress the expression of PD-L1 and inhibit the process of epithelial-mesenchymal transition (EMT). This is achieved by up-regulating the levels of *miR-200c* [237].

## Ultrasound-mediated targeted nanobubbles for liver cancer treatment: harnessing the synergistic effect of PD-L1 antibody and miRNA-mediated immunotherapy

Ultrasound-mediated targeted nanobubbles hold promise as a prospective vehicle for liver cancer treatment. The combination of PD-L1 antibody and *miR-424* within these NBs exhibits a synergistic effect, enhancing the effectiveness of anti-tumor immunotherapy. The combined immunotherapeutic effect of the anti-PD-L1 antibody and *miR-424* was assessed in a mouse model with hepatoma-transplanted tumors. The targeted NBs facilitated the delivery of the PD-L1 antibody to block the PD-1/PD-L1 signaling pathway and decrease the expression of PD-L1 in tumor cells. This resulted in an enhanced immune response mediated by T cells, leading to increased anti-tumor effects [238].

Researchers utilized NBs for targeted delivery of *miR-195* and Chlorine e6 (Ce6) to mouse models of hepatocellular carcinoma. They observed that *miR-195* expression disrupted the interaction between PD-1 and PD-L1, leading to an increase in the activity of cytotoxic T cells (CTLs) and enhanced immune response. Additionally, Ce6 was employed as a sonosensitizer to induce Sonodynamic Therapy (SDT) and initiate Immunogenic Cell Death (ICD) in tumor cells. The combination of SDT-induced immunogenic cell death and immune checkpoint blockade of PD-1/PD-L1 through *miR-195* upregulation resulted in the activation of a stronger antitumor immune response [239].

**Fig. 4:**
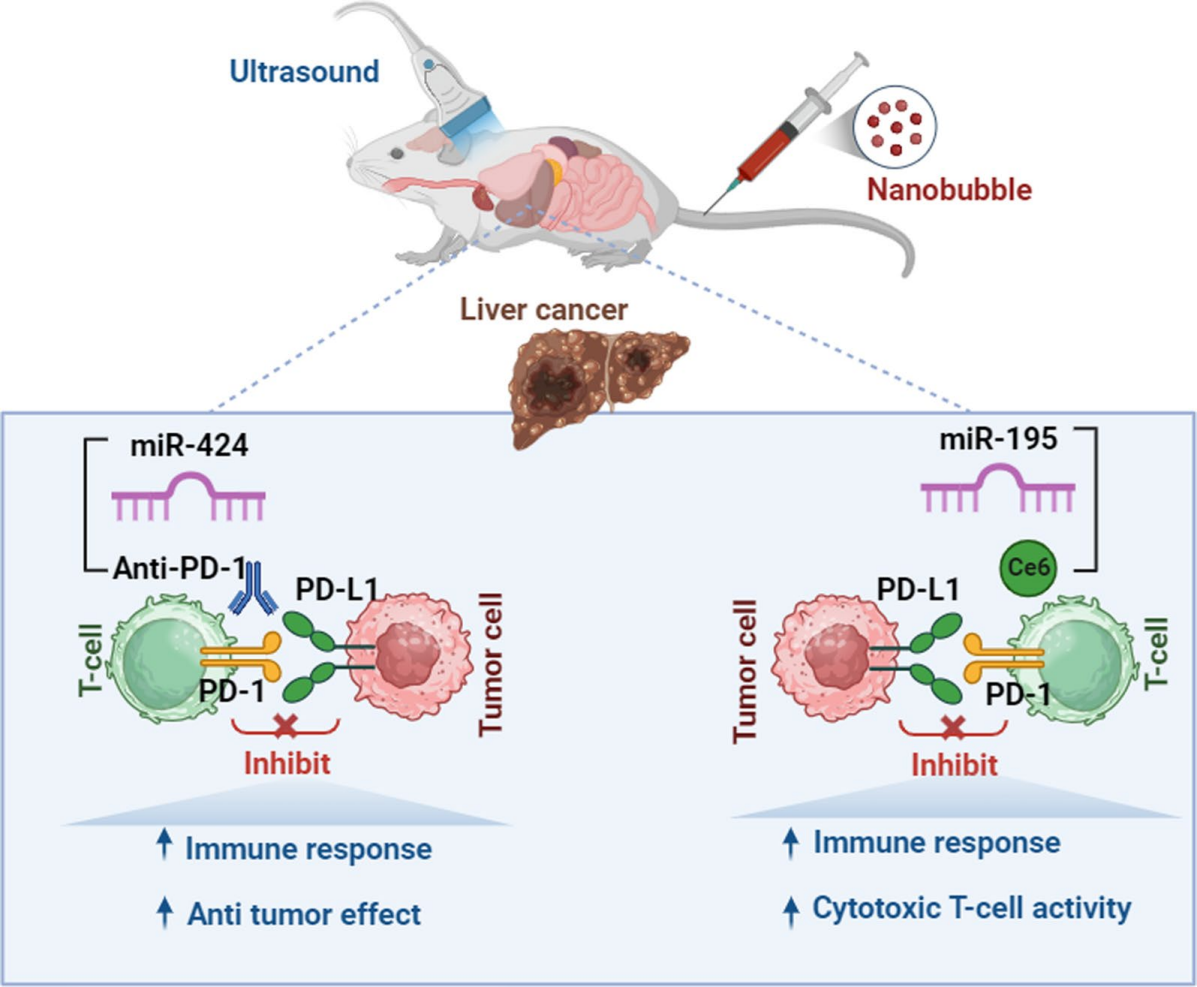
Ultrasound-mediated targeted nanobubbles for liver cancer treatment

Accordingly, the Fig. 4 illustrates the use of ultrasound-mediated targeted NBs as a potential therapeutic approach for liver cancer treatment.

## miRNA-mediated regulation immune checkpoints inhibitors in other diseases

Patients with chronic hepatitis B, liver cirrhosis, and hepatocellular carcinoma showed increased levels of PD-1 and decreased levels of *miR-138*. PD-1 expression was found to have a positive correlation with the hepatitis B virus (HBV) viral load, while *miR-138* showed a negative correlation. Through its interaction with the 3'-untranslated region of PD-1, *miR-138* inhibited the expression of PD-1, resulting in the downregulation of PD-1 in T cells and the upregulation of antiviral cytokines. These findings suggest that *miR-138* could potentially serve as a therapeutic target for the treatment of HBV infection [240].

Tuberculosis (TB) is a fatal contagious illness caused by the bacterium Mycobacterium tuberculosis (M. tuberculosis). The development of effective vaccines and treatments relies on the identification of reliable indicators of immune protection. A recent study discovered that severe cases of TB result in the increased activity of a protein called interferon regulatory factor 7 (IRF7) and its associated genetic markers. When IRF7 is genetically deactivated, it leads to more severe lung damage, higher levels of M. tuberculosis in the body, impaired differentiation of T cell subsets responsible for immune response, and increased production of pro-inflammatory cytokines. IRF7 plays a crucial role in maintaining the expression of PD-1/PD-L1, as well as the regulation of *miRNA-31*, which is influenced by the PD-1/PD-L1 pathway [241].

The impact of PD-1 in causing cardiac damage in mice was investigated and demonstrated that the use of a PD-1 inhibitor resulted in the polarization of macrophages towards the M1 phenotype, leading to cardiac damage. Moreover, the administration of a PD-1 inhibitor led to cardiac dysfunction and inflammation within the myocardium. Conversely, the PD-1 inhibitor facilitated M1 polarization and cardiac injury by modulating the signaling pathway of *miR-34a/KLF4*, ultimately inducing inflammation in the myocardium. These findings could enhance our understanding of the mechanisms underlying cardiac injury in cancer patients during immunotherapy. They also have the potential to identify novel targets for mitigating or treating cardiac injury in such individuals [242, 243].

The *circFLNA/miR-214-5p*/PD-1 signaling pathway has been identified as a novel mechanism involved in the regulation of Tregs in sepsis-induced acute respiratory distress syndrome (ARDS). In ARDS, there is an abnormal increase in *circFLNA* expression. However, depletion of *circFLNA* leads to an upregulation of CD4 + CD25 + Foxp3 + Tregs and a reduction in the inflammatory response associated with ARDS. Furthermore, the interaction between *miR-214-5p* and *circFLNA* was found to counteract the effects induced by *circFLNA* in ARDS. PD-1 was identified as a downstream target gene of *miR-214-5p* and was able to negate the regulatory effects of *miR-214-5p* on CD4 + CD25 + Foxp3 + Tregs and the inflammatory response [244].

Stimulation of PD-1 using PD-L1 Ig alleviates intestinal immune defense injury by activating the IL-10/*miR-155* pathway in response to intestinal ischemia–reperfusion (I/R) injury. PD-1, IL-10, and *miR-155* represent potential targets for mitigating intestinal barrier damage and modulating immune responses [245].

The *miR-155-5p* molecule plays a role in the programmed cell death of CD34 + cells in Myelodysplastic Syndromes (MDS) through the *RAC1/CREB/miR-15b* pathway, thereby suppressing the production of blood cells in the bone marrow. *miR-155-5p* can induce the expression of PD-L1, which is involved in this process. However, increasing the levels of miR-15b hampers this effect, thereby affecting the expression of PD-L1 [246].

Behcet's disease is characterized by a notable increase in the expression of both *miR-155* and TNF-α, while the expression of CTLA-4 is significantly reduced. Additionally, a negative correlation between *miR-155* and CTLA-4 expression was observed. Therefore, the expression of *miR-155* is elevated in Behcet's disease and is associated with the upregulation of TNF-α and downregulation of CTLA-4 genes [247].

Table 4 summarizes the current understanding of the role of miRNAs in regulating immune checkpoint inhibitors in various diseases. It includes information on the disease, the specific miRNA involved, the mechanism by which the miRNA regulates the immune checkpoint, and the effect of this regulation.

**Table 4:** miRNAs function in regulating immune checkpoint inhibitors in various diseases

Disease	miRNA	Mechanism	Effect
Chronic hepatitis B	*miR-138*	Targets of PD-1, inhibiting its expression	Upregulates antiviral cytokines
Tuberculosis	*miR-31*	Regulated by the PD-1/PD-L1 pathway	Influences the differentiation of T cell subsets and production of pro-inflammatory cytokines
Cardiac damage	*miR-34a*	Modulates the *miR-34a/KLF4* signaling pathway	Facilitates M1 polarization and cardiac injury
Sepsis-induced ARDS	*miR-214-5p*	PD-1 interaction	Abnormal increase in circFLNA expression
Intestinal ischemia–reperfusion injury	*miR-155*	Activates the IL-10/*miR-155* pathway	Mitigates intestinal barrier damage and modulates immune responses
Myelodysplastic Syndromes	*miR-155-5p*	Induces expression of PD-L1	Suppresses production of blood cells in bone marrow
Behcet's disease	*miR-155*	Upregulates TNF-α, downregulates CTLA-4	Elevated expression of *miR-155*, associated with TNF-α and CTLA-4 genes

## Conclusion

ICIs have revolutionized cancer treatment, but their efficacy can be limited by tumor immune evasion mechanisms. miRNAs are small non-coding RNAs that play a crucial role in regulating gene expression, including the expression of immune checkpoint molecules such as PD-1 and PD-L1. Recent studies have demonstrated that miRNAs can modulate tumor immune evasion and influence the response to ICIs.

The dysregulation of miRNAs, resulting in either upregulation or downregulation of ICIs expression, can significantly impact tumor growth, metastasis, and response to immunotherapy. The intricate interplay between miRNAs, ICIs, and the tumor microenvironment highlights the potential of miRNAs as therapeutic targets for cancer treatment.

We demonstrated the ability of miRNAs to modulate PD-1 and PD-L1 expression in various cancer types, including gastrointestinal cancers, hepatocellular carcinoma, glioblastoma, breast cancers, cervical, ovarian, and endometrial cancer, diffuse large B cell lymphoma, melanoma, bladder, and prostate cancer. The mechanisms by which miRNAs regulate ICIs expression are diverse and involve direct targeting of mRNA, acting as sponges for other miRNAs, and modulating signaling pathways related to their expression.

The therapeutic potential of miRNAs in cancer treatment is gaining increasing attention. Targeting miRNAs that upregulate PD-L1 expression, such as *miR-214, miR-105-5p, miR-502-5p, miR-194-5p, hsa_circ_0046523*, and *miR-124-3p*, could enhance the efficacy of immunotherapy by restoring antitumor immunity. Conversely, miRNAs that downregulate PD-L1 expression, such as *miR-140-3p, miR-30a-5p, miR-429*, and *miR-5590-3p*, could be used to sensitize tumors to immunotherapy.

The development of miRNA-based therapies for cancer is still in its early stages, but the potential of miRNAs to modulate PD-L1 expression and enhance antitumor immunity is promising.

In addition to cancer, miRNAs have also been shown to play a role in regulating immune checkpoints in other diseases, such as autoimmune diseases and infectious diseases. Further research into the role of miRNAs in these diseases may lead to the development of new therapeutic strategies.

Comprehensively, miRNA-mediated regulation of immune checkpoints represents a promising new approach to cancer immunotherapy. With further research, miRNA-based therapies may have the potential to revolutionize the treatment of cancer and other diseases.

## Data Availability

Agree.
